# Deciphering Mechanisms, Prevention Strategies, Management Plans, Medications, and Research Techniques for Strokes in Systemic Lupus Erythematosus

**DOI:** 10.3390/medicines11070015

**Published:** 2024-07-31

**Authors:** Ola A. Al-Ewaidat, Moawiah M. Naffaa

**Affiliations:** 1 Department of Internal Medicine, Ascension Saint Francis Hospital, Evanston, IL 60202, USA; ola.al.ewaidat@ascension.org; 2 Department of Cell Biology, Duke University School of Medicine, Durham, NC 27710, USA; 3 Department of Psychology and Neuroscience, Duke University, Durham, NC 27708, USA

**Keywords:** systemic lupus erythematosus, stroke, antiphospholipid syndrome, antiphospholipid antibodies, aspirin, statin, oral anticoagulant, biologic drugs

## Abstract

Systemic lupus erythematosus (SLE) is an autoimmune rheumatic condition characterized by an unpredictable course and a wide spectrum of manifestations varying in severity. Individuals with SLE are at an increased risk of cerebrovascular events, particularly strokes. These strokes manifest with a diverse range of symptoms that cannot be solely attributed to conventional risk factors, underscoring their significance among the atypical risk factors in the context of SLE. This complexity complicates the identification of optimal management plans and the selection of medication combinations for individual patients. This susceptibility is further complicated by the nuances of neuropsychiatric SLE, which reveals a diverse array of neurological symptoms, particularly those associated with ischemic and hemorrhagic strokes. Given the broad range of clinical presentations and associated risks linking strokes to SLE, ongoing research and comprehensive care strategies are essential. These efforts are critical for improving patient outcomes by optimizing management strategies and discovering new medications. This review aims to elucidate the pathological connection between SLE and strokes by examining neurological manifestations, risk factors, mechanisms, prediction and prevention strategies, management plans, and available research tools and animal models. It seeks to explore this medical correlation and discover new medication options that can be tailored to individual SLE patients at risk of stroke.

## 1. The Silent Threat: Stroke Risk in Systemic Lupus Erythematosus

Systemic lupus erythematosus (SLE) is a complex autoimmune disorder associated with chronic inflammation, organ damage, and diverse clinical symptoms such as joint pain and fatigue. Beyond the disease itself, patients face risks from medication side effects and thrombotic events, including deep vein thrombosis and pulmonary embolism, which can be life-threatening [1].

Cerebrovascular disorders, notably stroke, affect 5% to 15% of SLE patients, significantly impacting their health and well-being [2,3,4,5,6]. The heightened susceptibility to stroke underscores the need for targeted efforts to understand and manage these events within the context of SLE.

Stroke in SLE can manifest as ischemic or hemorrhagic, influenced by disease-specific factors and conventional risks like hypertension and smoking [7,8]. Comprehensive research and tailored interventions are crucial to address this complex interaction, optimize outcomes, and mitigate the debilitating effects of stroke in SLE patients.

Neuropsychiatric SLE (NPSLE) presents additional challenges due to its diverse and complex neurological manifestations. It commonly includes acute cerebrovascular diseases such as strokes and transient ischemic attacks, alongside seizures, cranial neuropathy affecting vision or facial function, and cognitive dysfunction impacting memory and problem-solving abilities [2,9,10,11,12].

Ischemic stroke, a severe complication of NPSLE, affects 3% to 20% of patients with SLE, indicating variability in study populations and diagnostic criteria [13,14,15]. Managing NPSLE requires a multidisciplinary approach involving rheumatologists, neurologists, and other specialists to address its complex symptoms and tailor treatments to individual patient needs.

The ten-year survival rate for SLE patients is 92%, highlighting the need to focus on cardiovascular risks, including stroke, which increases by 3% annually with age [16,17]. While numerous studies have explored stroke risk in SLE, focusing mainly on specific stroke subtypes, there remains a gap in comprehensive research covering all stroke categories in this population. Large-scale cohort studies are essential to evaluate long-term ischemic stroke risks and develop appropriate prophylactic strategies. Table 1 summarizes key findings from relevant studies on stroke prevalence among SLE patients.

The relationship between SLE and hemorrhagic strokes remains incompletely understood [7]. Ischemic strokes, which account for approximately 85% of cases, are caused by vascular blockages. In contrast, hemorrhagic strokes, though less common, are more prevalent in SLE patients over 50 with hypertension [16,32].

**Figure 1:**
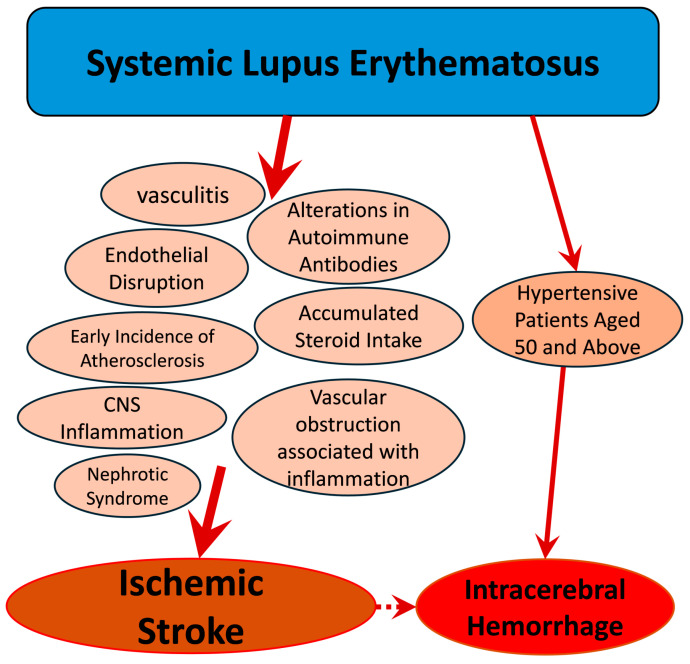
Flowchart illustrating the pathophysiological mechanisms of stroke in patients with SLE.

Several mechanisms contribute to the pathogenesis of ischemic strokes in SLE patients, including vasculitis, altered antibodies, endothelial dysfunction, cumulative steroid use, and early-onset atherosclerosis (Figure 1) [33,34,35,36]. Advanced age is a significant risk factor for atherosclerosis, leading to atherothrombotic strokes, the most frequent type of ischemic stroke [37,38]. Persistent thrombotic risk in SLE is exacerbated by neuroinflammation, characterized by oxidative stress, matrix metalloproteinase production, and microglial activation [39,40]. Increased occurrence of small vessel disease, microthrombi, and complement deposition in SLE patients may further contribute to inflammatory vascular occlusion, elevating stroke incidence [41,42].

Several studies suggest that common cardiovascular risk factors do not fully explain the increased stroke incidence in SLE patients [43]. Renal involvement in SLE may contribute to stroke risk [3]. Ischemic stroke can be an early sign of nephrotic syndrome (NS) and should be considered when excluding common ischemic stroke causes, particularly in the presence of pre-existing glomerular disease [44].

In a study of SLE patients, 65% of those with stroke had renal involvement [45]. NS, often arising from glomerular diseases, is linked to venous thromboembolism (VTE) and, less frequently, arterial thromboembolism (ATE) [46]. NS increases the risk of functional dependence after ischemic stroke, suggesting the need for intensive treatment and rehabilitation [47]. While ATE and acute ischemic stroke are rare in NS, they can occur in young patients and may be the first indication of NS. Data on ATE risk factors in NS are scarce and primarily derived from VTE data [48].

There are cases of young patients with new-onset lupus nephritis experiencing acute stroke without traditional SLE-related stroke risk factors, highlighting the need for further research to better understand stroke risk in SLE patients [49]. One case involved a young woman with lupus nephritis who had a stroke caused by large-vessel vasculitis, confirmed by vessel wall MRI [50].

This review offers current insights into stroke in SLE patients and suggests future research directions for personalized medication options. It covers prediction, prevention, management strategies, and tools to understand the pathological link between stroke and SLE, aiming to improve treatment outcomes. This foundational knowledge supports tailored interventions to reduce stroke risk and enhance holistic care for individuals with SLE.

## 2. Antiphospholipid Syndrome and Neurological Implications: Unraveling the Stroke Connections

Antiphospholipid syndrome (APS) was initially identified through the discovery of the lupus anticoagulant autoantibody, which binds to phospholipids and proteins associated with cell membranes, and its correlation with other autoimmune disorders [51]. APS is now characterized as an autoimmune disorder marked by venous or arterial blood clots and/or complications during pregnancy in individuals with antiphospholipid antibodies (aPLs) [52]. It can manifest as either a primary disease or a secondary condition, often linked to autoimmune disorders like SLE, rheumatoid arthritis, Sjogren’s syndrome, or systemic sclerosis [53,54]. APS is characterized by recurring clotting events, miscarriages, and low platelet counts, along with the persistent presence of antiphospholipid antibodies, including anticardiolipin, lupus anticoagulant, and anti-beta 2-glycoprotein I (anti-β2GPI). Approximately 36% of APS patients also have SLE, increasing the risk of aPL-mediated cardiovascular events [51].

APS is associated with a spectrum of brain-related events, encompassing both clot-related and non-clot-related conditions. Clot-related events, such as stroke and transient ischemic attacks (TIAs), are observed in individuals with APS [55]. Additionally, non-clot-related events, including seizure disorders and cognitive dysfunction, are part of the neurological manifestations associated with APS [55].

Both APS and aPLs play a role in contributing to brain bleeding events, a phenomenon similarly observed in SLE [56,57,58]. While intracranial bleeding occurs in approximately 0.4% of SLE patients [59], subarachnoid bleeding is also identified as a potential complication in SLE [60]. It is noteworthy that subarachnoid bleeding, though rare, can still occur in individuals with APS [61].

APS is a well-established risk factor for stroke and other NPSLE syndromes. Cerebral ischemia cases are associated with secondary APS, suggesting a mechanistic role [62,63,64,65]. Ischemic stroke was initially attributed to a heightened clotting tendency, but other mechanisms unrelated to clot formation, such as vascular abnormalities or heart valve issues, also play a role [66,67,68,69].

The connection between strokes and antiphospholipid syndrome (APS) in individuals under 45 years old has been established through research, indicating that over 20% of strokes in this age group are linked to APS [70]. Moreover, in patients with secondary APS associated with systemic lupus erythematosus (SLE) and those with primary APS, coupled with vascular risk factors, there is an increased risk of blood clotting [71]. The debate arises when considering the role of traditional vascular risk factors versus the presence of circulating antiphospholipid (aPL) antibodies in the accelerated development of atherosclerosis observed in APS patients. Studies explore whether high blood pressure, which is more prevalent in SLE and APS compared to the general population, acts as the sole independent risk factor for arterial complications, particularly stroke, in APS [72].

Despite approximately 10% of cerebrovascular events occurring in younger populations without a clear explanation, aPLs are recognized as significant risk factors for ischemic stroke and recurrent clot-related events [73]. In individuals with SLE, lupus anticoagulant emerges as a more potent predictor of clot formation compared to anticardiolipin antibodies [74]. A decade-long study established a robust association between the presence of lupus anticoagulant at the outset and subsequent intracranial clot formation in SLE patients [75].

**Figure 2:**
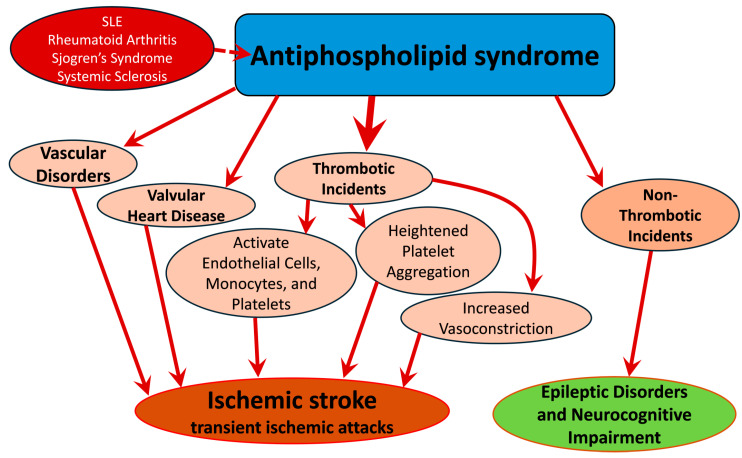
Flowchart illustrating the pathophysiological mechanisms of stroke in patients with APS.

The primary mechanism underlying nervous system involvement in APS is associated with blood clot formation, with aPLs playing a pivotal role [76]. These antibodies activate endothelial cells, monocytes, and platelets, inducing a pro-clotting state, increased platelet aggregation, and heightened blood vessel constriction (Figure 2) [77]. Evidence suggests that aPLs activate the lining of blood vessels in the brain, promoting pro-clotting activity and causing damage to nerve cells [77]. The interaction between aPLs and the inner surfaces of blood vessels leads to the upregulation of adhesion molecules and the release of proinflammatory chemicals [78]. However, the precise mechanisms by which these antibodies induce blood clot formation remain unclear [79]. Importantly, individuals with aPLs face a 5-fold higher risk of stroke or TIA compared to those without aPLs [73].

Patients with SLE and APS who exhibit a triple-positive aPL antibody profile are identified as a high-risk group for thrombotic events, including strokes [80]. This heightened risk is particularly pronounced in individuals with multiple positive autoantibodies, notably those classified as “triple positive” [81]. The presence of a triple aPL antibody profile in patients with SLE and APS significantly predisposes younger individuals (under 50 years of age) to an elevated risk of stroke and recurrent thromboembolic events [82,83].

Direct oral anticoagulants (DOACs), such as rivaroxaban, are not recommended for patients with triple-positive APS due to their association with a high risk of recurrent thrombotic events and the lack of comprehensive studies confirming the safety and efficacy of other DOACs in this population [84]. The management of secondary stroke prevention in patients with SLE and a triple aPL antibody profile is complex. It requires a delicate balance between mitigating the risks of bleeding and preventing recurrent thrombotic events. Therefore, each case necessitates a personalized approach that carefully weighs the risks and benefits of various therapeutic options within a multidisciplinary framework, as demonstrated in our patient’s case.

Optimizing stroke management outcomes in patients with APS requires physicians to consider factors such as cardiac manifestations, inflammation, and accelerated atherosclerosis. Although the precise pathophysiology of APS remains unclear, genetics and triggers such as infections are believed to play crucial roles in disease onset. Emerging prevention guidelines emphasize personalized treatment strategies, necessitating comprehensive analysis of test results and collaborative input from specialties such as rheumatologists, neurologists, and hematologists in managing stroke in APS patients.

## 3. Using Biomarkers and Autoantibodies to Predict Stroke Risk in Systemic Lupus Erythematosus and Develop Individualized Management Plans

SLE significantly increases the risk of premature atherosclerosis, with a 6-fold higher cardiovascular risk compared to the general population [85]. The gradual onset of SLE manifestations complicates early diagnosis [86]. Therefore, identifying biomarkers that reflect ongoing inflammation driving atherosclerotic plaque formation is crucial for guiding therapeutic interventions in SLE. Studies have identified piHDL, leptin, and sTWEAK, along with factors like age and diabetes, as part of the “Predictors of Risk for Elevated Flares, Damage Progression, and Increased Cardiovascular Disease in Patients with SLE (PREDICTS)” profile. This profile enhances the identification of SLE patients at risk for subclinical atherosclerosis progression, including carotid plaque and intima–media thickness (IMT) progression [87,88,89].

Recent research underscores immune cells and cytokines as potential biomarkers for early atherosclerosis in SLE. Studies show correlations between molecules such as interleukins, interferons, and immune cell markers (e.g., VCAM-1, VAP-1, β2-microglobulin) and atherosclerosis/CVD. However, clinical application remains inconclusive due to complex interactions and inconsistent findings across studies. Validating these biomarkers and developing effective therapeutic strategies are essential for preventing atherosclerosis in SLE patients. Recent reviews provide comprehensive insights into the current understanding and advancements in atherosclerosis biomarkers in SLE [90].

The diagnosis of neurological involvement in SLE requires specific clinical and laboratory criteria [91]. Although thrombosis or cerebrovascular accidents (CVAs) are not part of the diagnostic criteria for SLE, the immunological activation associated with the disease increases the risk of clotting, making stroke a plausible complication in SLE patients [92]. This insight underscores the importance of early implementation of medication plans to prevent stroke in SLE patients. These plans may involve the use of existing medications or the development of new treatments specifically designed to address the risk factors associated with stroke in SLE.

Antinuclear autoantibodies (ANAs) have been correlated with intracranial arterial stenosis in SLE patients experiencing ischemic strokes, an association attributed to endothelial cell dysregulation (Table 2) [93,94]. French national guidelines recommend testing for anti-SS-A antibodies during the second-line assessment of stroke in adults under 55 when no clear etiology is found. However, the presence of anti-SS-A antibodies in these patients is rare, occurring in only 1–2% of cases [95]. The inflammatory response in the brains of SLE patients with strokes, linked to anti-double-stranded DNA (anti-dsDNA) autoantibodies, is characterized by elevated levels of IL-1β and IL-6 [96]. Despite these findings, current assays for detecting ANAs and anti-dsDNA lack reliability and interpretability [97]. These tests cannot currently be assessed in the context of studying stroke in SLE patients, as their reproducibility and effectiveness have not yet been validated.

Anticardiolipin autoantibodies (aCLs) exhibit a significant presence in various diseases, including SLE, owing to their specific affinity for cardiolipin. However, their relationship with stroke, a common cerebrovascular event, is multifaceted [98]. The prevalence of aCL among individuals experiencing thrombotic cerebrovascular events varies widely, ranging from 6.8% to 46% [98,99,100,101]. It is important to note that not all studies confirm the persistence of aCL positivity beyond the initial 6–12 weeks from sampling, suggesting potential fluctuations in antibody levels over time.

Research has aimed to elucidate the specific role of aCL subtypes, particularly IgM and IgG, in stroke pathology. Some findings suggest a correlation between any titer of IgM subtype aCL and stroke incidence, while others propose that only high titers of IgM aCL are associated with increased stroke risk [102,103] (Table 2). Notably, the presence of IgG aCL does not appear to predict the recurrence of thrombosis, indicating a differential impact of aCL subtypes on thrombotic events [104].

Despite ongoing research efforts, the optimal treatment strategy for individuals with aCL-related stroke remains uncertain. Current literature discusses the potential benefits of high-dose warfarin alone or in combination with low-dose aspirin, yet definitive conclusions are lacking (Table 3) [105]. Nevertheless, emerging evidence suggests that low-dose aspirin could hold promise as a preventive measure for stroke in SLE patients, underscoring the importance of tailored treatment approaches in this population [106].

The involvement of both IgM and IgG subtypes of anti-β2GPI autoantibodies in the pathogenesis of antiphospholipid syndrome (APS) highlights their crucial role in the development of strokes and cerebral vein thrombosis [107,108,109]. Particularly noteworthy is the persistence of these antibodies in stroke patients even upon retesting, indicating their enduring presence and potential clinical significance [110].

Specifically, persistent IgG anti-β2GPI has been identified as an independent risk factor for recurrent stroke, emphasizing its pivotal role in the management and prognosis of stroke cases (Table 2) [111]. In contrast, IgM anti-β2GPI has been independently associated with strokes, suggesting its potential as a more reliable predictor compared to IgG anti-β2GPI [112]. However, the interpretation of IgM anti-β2GPI’s role is complicated by findings from one study suggesting it might be associated with a reduced risk of stroke, necessitating further investigation (Table 2) [102]. The prevalence of anti-β2GPI in stroke patients varies considerably, with estimates ranging from 5% to 24%, though certain studies did not include retesting to validate these estimates [113,114].

**Table 2:** Autoantibodies used to predict stroke risk in SLE.

Autoantibody	Findings	Reference
Antinuclear autoantibodies (ANAs)	ANAs are linked to intracranial arterial stenosis in SLE patients with ischemic strokes via endothelial cell dysfunction.	[93,94]
Anti-double-stranded DNA autoantibodies (anti-dsDNA)	In SLE stroke patients, brain inflammation linked to anti-dsDNA antibodies shows increased IL-1β and IL-6 levels.	[96]
Anticardiolipin autoantibodies (aCLs)	Research on aCLs subtypes (IgM and IgG) in stroke suggests that elevated levels of IgM aCL may increase stroke risk, while IgG aCL does not predict thrombosis recurrence, indicating subtype-specific effects on thrombotic events.	[102,103,104]
IgG anti-β2GPI	Persistent IgG anti-β2GPI is crucial for stroke management and prognosis, as it is a key risk factor for recurrent stroke.	[111]
IgM anti-β2GPI	IgM anti-β2GPI is independently linked to strokes and may be a better predictor than IgG anti-β2GPI. Yet, one study suggests it could reduce stroke risk, complicating its role.	[102,112]
Lupus anticoagulant (LAC)	LAC binds to phospholipids, significantly increasing stroke risk, often impacting critical brain regions like the left MCA and left superior cerebellar artery.	[114,115,116]

Regarding treatment strategies, there was no significant difference in recurrence rates between stroke patients with isolated IgM APS positivity treated with antiplatelet agents and those treated with Vitamin K antagonists (VKAs) like warfarin [117]. However, within the subgroup of patients positive only for IgM, a higher recurrence rate was observed in the antiplatelet treatment group, underscoring the importance of tailored treatment approaches aligned with individual patient profiles [117].

Lupus anticoagulant (LAC) is recognized for its ability to bind to phospholipids, thereby emerging as a significant autoantibody linked to an elevated risk of stroke (Table 2) [114,115]. Strokes associated with LAC often present in extensive areas of the brain, affecting critical regions such as the left middle cerebral artery (MCA) and the left superior cerebellar artery [116]. 

In the domain of managing thrombo-inflammatory disorders like APS, an emerging therapeutic avenue involves targeting platelet surface receptors or their intracellular signaling pathways using antiplatelet agents such as aspirin [118]. Despite the potential benefits of anticoagulation therapy for stroke in the presence of LAC, it is crucial to acknowledge the limited number of studies investigating the incidence of major bleeding associated with such treatment [105]. This underscores the pressing need for further research aimed at comprehensively evaluating the safety and efficacy of anticoagulant therapy in the context of LAC-associated stroke management.

Cerebral venous thrombosis (CVT) represents a rare yet potentially severe condition that can lead to stroke in individuals spanning various age groups, including children, adults, and even newborns [119]. The pathophysiological mechanisms underlying CVT are complex, involving a multifaceted interplay of immunological factors. Notably, the presence of both aCL IgA and anti-β2GPI IgA antibodies has been identified within the context of CVT [120]. Furthermore, recent investigations have shed light on the potential role of antiphosphatidylserine/prothrombin antibody (aPS/PT) IgG as a biomarker for APS and its association with stroke [121]. While direct evidence linking aPS/PT IgG or aPS/PT IgM to CVT is currently lacking, the observed correlation of aPS/PT IgG with LAC suggests its potential involvement in the pathogenesis of CVT [120]. Exploring the roles of these autoantibodies may reveal new therapeutic targets for managing CVT complications in SLE.

The landscape of arterial thrombotic events linked to non-criteria aPL is characterized by complexity and multifaceted interactions. Within this landscape, IgA anti-β2GPI stands out as a significant variant, with a prevalence of 20% among stroke patients, as indicated by a single serology measurement [122]. This observation underscores the importance of IgA anti-β2GPI, suggesting its potential to supersede conventional risk factors like hypertension or dyslipidemia [122,123,124]. However, it is essential to recognize the limitations inherent in some data, which may not definitively establish a direct association with stroke [125].

In contrast to other non-criteria aPLs, aPS/PT IgG antibodies exhibit an independent correlation with stroke or mortality in patients with TIAs [125]. This highlights the distinct pathogenic mechanisms and clinical implications associated with different aPL subtypes. Therefore, understanding and acknowledging variations in the prevalence and clinical significance of these antibodies is paramount.

The role of aPLs in recurrent ischemic strokes (RISs) remains uncertain despite their association with thrombotic events, particularly in young patients. A systematic review and meta-analysis found that aPL positivity did not significantly correlate with RIS in adults [126]. Subgroup analyses, considering age under 50 years, aPL type, ethnicity, repeated testing, and data characteristics, did not show an increased risk of RIS [126]. Therefore, current evidence suggests that persistent aPL positivity and newer aPL tests in ischemic stroke patients do not reliably predict higher rates of RIS [110].

Factors such as sample size, comorbidities, inter-assay agreement, and the necessity for retesting play pivotal roles in accurately interpreting and contextualizing the findings related to aPL-associated thrombotic events. Considering these factors ensures a comprehensive understanding of the intricate relationship between non-criteria aPLs and arterial thrombotic events, ultimately guiding clinical decision-making and management strategies through the selection of the most suitable medications for affected patients.

SLE is linked to an elevated risk of venous thromboembolism (VTE) and arterial thromboembolism (ATE). Thrombophilic defects, including protein C and S deficiencies, antithrombin III deficiency, and genetic mutations such as factor V Leiden and prothrombin G20210A, are prevalent in SLE patients. Specifically, factor V Leiden and prothrombin G20210A mutations increase VTE risk by 20- and 30-fold, respectively, without impacting ATE risk [127].

Inherited thrombophilia, defined as a genetic predisposition to VTE typically manifesting in individuals under 45 and often recurring without clear cause, has a debated role in arterial thromboses like ischemic stroke and myocardial infarctions [128]. Thrombophilia screening is advised for young patients with spontaneous or atypical thromboses, recurrent arterial events, or a significant family history, as it can lead to crucial, life-saving treatments [129].

Conversely, inherited thrombophilias such as factor V Leiden and prothrombin G20210A are associated with an increased risk of arterial ischemic stroke in adults, although further research is required to understand the clinical management implications [130]. While thrombophilic defects are likely to increase VTE risk in SLE patients with aPLs, their potential role in stroke development remains uncertain. 

Several models have been developed to predict CVD in SLE, but they often underestimate risk when using general population algorithms. SLECRISK, a novel SLE-specific tool, was created to provide a more accurate estimate of CVD risk in SLE patients [131].

SLECRISK extends the American College of Cardiology/American Heart Association (ACC/AHA) model [43], incorporating SLE-related variables to better detect moderate- to high-risk for major adverse cardiovascular events (MACEs). It addresses the shortcomings of existing models by including factors commonly assessed in clinical practice, thus improving the identification of SLE patients needing preventive and risk-modifying therapy [131].

Internally validated, SLECRISK shows greater sensitivity than traditional CVD tools for predicting moderate- and high-risk for MACEs over ten years. It includes routinely collected risk factors, making it particularly useful for identifying higher risk in young females with severe SLE activity. If externally validated, SLECRISK could be integrated into SLE management guidelines to support early decision-making for the primary prevention of CVD [131].

## 4. Strategies for Stroke Prevention and Management in Systemic Lupus Erythematosus: From Anticoagulation to Novel Approaches

The clinical presentation of SLE is highly variable, reflecting the diverse underlying mechanisms of the disease. This variability has contributed to the limited success of numerous clinical trials aimed at effectively treating and curing SLE without adverse effects. Presently, there is no definitive curative therapy available for SLE. Standard treatment protocols typically involve a combination of corticosteroids, immunosuppressive agents, antimalarial drugs such as hydroxychloroquine, and non-steroidal anti-inflammatory drugs (NSAIDs) [132].

Regrettably, both individually and in combination, these conventional treatments lack specificity and are associated with a spectrum of undesirable side effects, including an elevated risk of severe infections [133]. Consequently, there is a pressing need within the healthcare community to develop innovative therapeutic strategies for managing SLE. These novel approaches are aimed at enhancing the quality of life and improving the long-term survival prospects of individuals with SLE by tailoring medication regimens to individualized needs and considerations.

In the mid-20th century, the four-year survival rate for patients with SLE was a mere 50% [134]. However, substantial improvement has been observed in recent decades, with the fifteen-year survival rate now being approximately 85% [135,136]. This advancement signifies considerable progress over the past several decades. Nevertheless, traditional treatments, including prednisolone, hydroxychloroquine, and immunosuppressives, continue to face challenges in effectively managing the disease, and some SLE patients still experience premature mortality. Furthermore, conventional therapies are associated with drug toxicity, which can lead to irreversible organ damage [137,138].

To mitigate CVEs in individuals with SLE, it is crucial to address modifiable risk factors such as dietary habits, physical activity, and comorbid conditions. Initiating aspirin is advisable, with clopidogrel serving as an alternative in select cases (Table 3) [139]. The administration of low-dose aspirin is pivotal for the primary prevention of thrombosis in SLE patients with aPL, particularly when other prothrombotic factors are present [3]. Therefore, for the primary prevention of stroke, aspirin is recommended alongside the management of cardiovascular risk factors, including hypertension, diabetes, tobacco use, and hyperlipidemia [140]. Although some meta-analyses indicate a significant reduction in the risk of initial thrombotic events, they also report a noteworthy increase in bleeding risk [141].

Hydroxychloroquine, a cornerstone of SLE therapy, has been shown to significantly reduce the risk of cardiovascular events [142,143,144]. Considering the progression of atherosclerosis and the anti-inflammatory effects of statins in SLE patients is warranted, particularly for those with dyslipidemia (Table 3) [3].

**Table 3:** Medications used to prevent and manage stroke in individuals with SLE.

Medications	Structure	Medication Usage in Preventive and Management Strategies	Reference
Aspirin	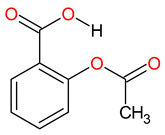	- Low-dose aspirin may help prevent stroke in SLE patients, emphasizing the need for personalized treatment. - Low-dose aspirin is key for preventing thrombosis in SLE patients with aPL, particularly with additional prothrombotic factors. - Aspirin is recommended to prevent stroke and manage cardiovascular risks like hypertension, diabetes, smoking, and hyperlipidemia.	[3,106,140]
Clopidogrel	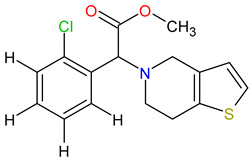	- Clopidogrel and aspirin may be considered for minor stroke or TIAs, despite limited studies on SLE and aPL. - To reduce cardiovascular events in SLE patients, start aspirin with clopidogrel as needed.	[139,145]
Warfarin	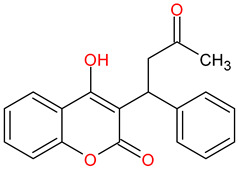	- Studies suggest benefits of high-intensity warfarin in APS, yet some limitations exist. - Recommend long-term warfarin for SLE patients with APS-related ischemic stroke. - Combining aspirin and warfarin for primary prevention is discouraged due to bleeding risk.	[105,146,147,148]
Statins (e.g., atorvastatin)	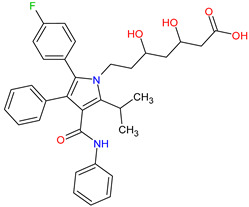	- Given statins’ benefits in SLE and atherosclerosis, focusing on dyslipidemic patients is crucial. - Low-dose aspirin or clopidogrel is typically prescribed for seronegative APS patients with arterial thrombotic events, with consideration given to statins if tolerated.	[3,149]
Rivaroxaban	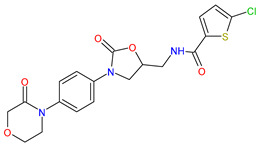	An ongoing trial compares high-intensity oral rivaroxaban and warfarin in APS patients with or without SLE, who have a history of stroke.	[150]
Prednisolone	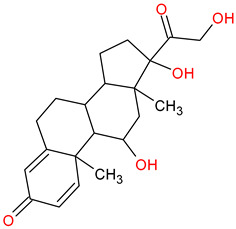	Prednisone’s potential to accelerate atherosclerosis in SLE patients necessitates aggressive stroke risk factor intervention.	[36]
Hydroxychloroquine	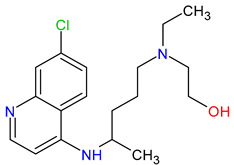	Hydroxychloroquine reduces cardiovascular event risk in SLE therapy.	[142,143,144]
Cyclophosphamide	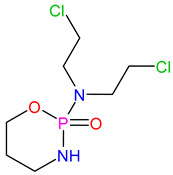	Immunosuppressive therapies, such as cyclophosphamide, manage stroke in lupus patients by reducing systemic inflammation.	[13,151]
Methotrexate (MTX)	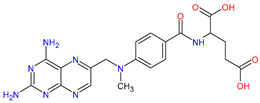	Monitoring of MTX-induced hyperhomocysteinemia and homocysteine levels following drug initiation in patients with SLE who are at high cardiovascular risk, such as for stroke.	[152]
Rituximab (PDB: 4KAQ)	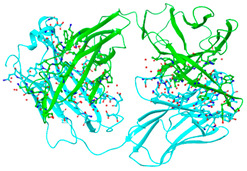	Rituximab mitigates stroke in lupus patients through systemic inflammation reduction.	[13,151,153]
Belimumab (PDB: 5Y9K)	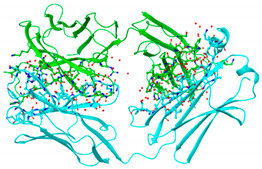	Belimumab reduces the risk of stroke in lupus patients by decreasing systemic inflammation.	[154]
Anifrolumab (PDB: 4QXG)	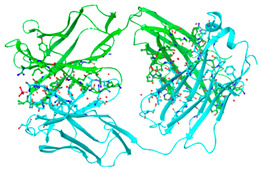	Anifrolumab may reduce the risk of stroke in lupus patients by attenuating systemic inflammation.	[155]

The approach to secondary prevention and treatment of stroke is generally consistent between patients with SLE and those without, except in cases where there is a history of recurrent stroke or APS. In such instances, anticoagulation therapy is warranted, potentially involving a higher international normalized ratio (INR) target if antiplatelet agents are not used concurrently [156]. The use of direct oral anticoagulants (DOACs) is typically not recommended and may pose a greater risk of major bleeding compared to warfarin [84]. Currently, an ongoing clinical trial is comparing the efficacy of high-intensity oral rivaroxaban versus high-intensity warfarin in patients with APS, with or without coexisting SLE, who have previously experienced a stroke (Table 3) [150].

For patients with APS or sustained high antiphospholipid antibody (aPL) titers, chronic oral anticoagulation therapy is warranted [140]. Anticoagulation is the primary modality for preventing stroke recurrence in high-risk SLE patients or those with secondary APS [8]. Although studies indicate the advantages of high-intensity warfarin in APS, certain limitations are noted [146]. Experts recommend the prolonged use of anticoagulation for SLE patients with ischemic stroke-secondary APS [147]. For minor stroke or TIAs, aspirin and clopidogrel may be considered, even though specific studies in the context of SLE and aPL are limited [145].

In cases of inconsistent positive tests for APS, it is prudent to perform an assessment for other autoimmune diseases, particularly SLE, as individuals with both APS and SLE face an increased risk of stroke [62]. The combined use of aspirin and warfarin for primary prevention is not recommended due to the elevated risk of bleeding. Multiple organizations, including the 13th International Congress on Antiphospholipid Antibodies and the European League Against Rheumatism, advocate for aspirin use in individuals with high-risk antiphospholipid profiles, those with other thrombotic risk factors, and those with SLE [149].

For patients with seronegative APS experiencing arterial thrombotic events, treatment typically involves low-dose aspirin or clopidogrel, with the potential addition of a statin if it can be tolerated [149]. Moreover, lifelong use of DOACs has been considered in patients with IgA anti-β2GPI antibodies [157]. Additionally, some studies suggest including Sneddon’s syndrome—a rare non-inflammatory thrombotic vasculopathy—in the spectrum of neurological manifestations associated with APS and SLE. This proposition is based on findings that over 40% of patients diagnosed with Sneddon’s syndrome have aPL. The syndrome is characterized by ischemic cerebrovascular disease, often accompanied by livedo reticularis [158].

The utilization of antithrombotic drugs poses a risk factor for hemorrhagic stroke. However, limited studies have investigated the correlation between hemorrhagic stroke and antithrombotic use in patients with SLE [159]. Further research is required to provide additional insights into this correlation. A recent study found that the association between anticoagulants and hemorrhagic stroke in SLE patients was non-significant [30]. Prophylaxis against thrombotic events in patients with SLE primarily involves the administration of antiplatelets or anticoagulants [106,160]. However, it is important to note that this treatment is associated with an increased risk of hemorrhagic stroke [28].

The introduction of biologic drugs, which are derived from or contain components of living organisms, has significantly enhanced outcomes and quality of life for patients with autoimmune rheumatic diseases such as rheumatoid arthritis, psoriatic arthritis, and ankylosing spondylitis [161]. However, relatively few biologic drugs have demonstrated efficacy in the routine management of SLE. B-cell blocking therapies, or B-cell depletion therapies (BCDTs), are crucial for treating autoimmune diseases and B-cell malignancies. These therapies target B cells, which are white blood cells essential for cytokine production, antigen presentation to T cells, and inflammation regulation. The goal is to eradicate harmful B cells while preserving beneficial B-cell immunity [162]. B-cell blocking therapies approved for use in SLE have shown promise in controlling disease activity, reducing the need for steroids [163], and slowing damage progression in open-label extensions of randomized controlled trials [164,165]. Unfortunately, not all patients respond equally to these drugs. For example, rituximab has a response rate of approximately 70 to 80% [153,166], while belimumab provides at least a 50% improvement in roughly half of the patients treated in the OBSErve Study [167]. Furthermore, some patients who initially respond well to rituximab may experience relapses [168], while others may develop allergic reactions [169].

The development of biologic therapies for systemic lupus erythematosus (SLE) significantly lags behind the advancements achieved in other rheumatological conditions. Currently, only two biologic agents, belimumab and anifrolumab, are approved for the treatment of lupus patients [170]. Analyses of trials involving rituximab, belimumab, and anifrolumab have underscored the critical importance of precise study design and the careful selection of appropriate outcome measures to reveal the potential advantages of new biologic drugs for SLE [165]. Furthermore, due to the diverse nature of SLE, the positive effects of certain drugs may not manifest uniformly across the entire population of lupus patients but may be confined to specific subgroups. Lessons learned from lupus trials should now inform the development of more effective Phase II and III studies [165]. Ultimately, this will pave the way for the emergence of enhanced biologic drugs that can make a substantial contribution to lupus therapy and help prevent the occurrence of cardiovascular events, especially strokes, in patients. Studying this aspect during clinical trials may help develop biologic drugs that prevent the severity of SLE and minimize the occurrence of strokes.

Several obstacles have impeded the progress of biologic drug development for SLE, leading to a continued reliance on conventional immunosuppressants like cyclophosphamide (Table 3). These conventional treatments carry the burden of various adverse effects and an increased risk of damage accumulation, especially when corticosteroids are involved [171].

## 5. Neuropsychiatric Symptoms in Systemic Lupus Erythematosus: Insights from Mouse Models for Developing New Therapeutic Options for Managing Stroke Incidence in SLE Patients

Investigating the complex pathophysiology of SLE through primary lupus mouse models presents significant challenges. One of the primary obstacles is the anatomical and immunological differences between mice and humans, which complicates the extrapolation of findings. Additionally, the expression of SLE varies significantly among different mouse models, making it difficult to generalize results to the human condition. Consequently, no single mouse model can fully replicate human SLE [172,173].

Despite these limitations, murine models offer valuable insights into the pathogenesis of SLE. They serve as crucial tools for exploring innovative treatment approaches [174]. Each mouse model has unique strengths and weaknesses making it well suited to specific preclinical research objectives. For instance, some models may better mimic particular aspects of human SLE, allowing for targeted investigations of disease mechanisms and potential therapies. However, it is important to note that research on stroke occurrence in the context of SLE remains limited.

The NZBWF1 mouse model is well known for its spontaneous development of SLE and is regarded as the most established and widely used classical model for SLE research (Table 4) [174]. This model exhibits key characteristics such as systemic autoimmunity, hemolytic anemia, proteinuria, and immune complex glomerulonephritis, which typically emerge around 5 to 6 months of age. Notably, disease severity is more pronounced in female mice, partly due to estrogen levels, resulting in a shorter lifespan for females compared to males (Table 4) [174,175,176,177].

Leveraging diverse mouse models with spontaneous or inducible lupus-like diseases is essential for comprehensively studying human disease and developing innovative treatments. While prior investigations have predominantly focused on genetically susceptible animals such as MRL/lpr mice for studying spontaneous neuropsychiatric SLE [178], inducible wild-type lupus models, like PIL mice, devoid of a specific genetic background, offer a more precise replication of clinical manifestations (Table 4) [180]. These models hold promise for advancing our understanding of the neuroimmune mechanisms underlying neuropsychiatric SLE, potentially unraveling intricate molecular and cellular pathways and laying the groundwork for novel therapeutic interventions. 

As early as week 2, PIL mice demonstrate significant elevations in cytokine and chemokine expression within the brain, preceding the onset of behavioral deficits [180]. This observation suggests a plausible link between the initiation of depressive-like behavior in PIL mice and cytokine overexpression.

The compromised brain function observed in PIL mice likely arises from cytokine dysregulation, resulting in blood–brain barrier impairment, IgG deposition, activation of glial cells, and neuronal damage. These findings underscore the importance of PIL mice as a valuable model for neuropsychiatric SLE research and underscore the critical roles played by glial cells in its pathogenesis [180]. Targeting glial cells may emerge as a promising therapeutic avenue for neuropsychiatric SLE, shedding light on the molecular and cellular mechanisms through which SLE contributes to the occurrence and recurrence of strokes.

Exploring NPSLE in humans is challenging due to the difficulties associated with brain biopsies. While postmortem histological analysis is an option, the rapid decomposition of brain tissue significantly affects the reliability of findings. Consequently, animal models are indispensable for unraveling the pathogenic mechanisms of NPSLE [183].

Commonly employed NPSLE animal models, such as NZB/W F1 and MRL/lpr mice, spontaneously exhibit neuropsychiatric symptoms, including cognitive dysfunction [184,185], anxiety, depression-like behavior [185,186,187], and reduced locomotor activity [177]. However, these models rely on inbred mouse strains, display a delayed and inconsistent onset of SLE-related symptoms, and progress slowly (Table 4) [179]. The heterogeneity and mild presentation of neuropsychiatric symptoms limit the applicability of these models in NPSLE research. Additionally, these genetically predisposed models do not fully capture the impact of environmental factors on NPSLE pathogenesis. 

Despite limitations, these models are valuable for probing stroke onset in SLE, elucidating molecular mechanisms, and pinpointing markers for stroke prevention and management.

Affective deficits are prevalent neuropsychiatric disturbances in NPSLE, significantly impacting patients’ quality of life [188]. Various genetically predisposed mouse models of NPSLE also manifest affective disorders. For instance, the MRL/lpr strain exhibits depression-like behavior and cognitive deficits but lacks anxiety-like behavior [185]. Additionally, NZB/W F1 mice display congenital abnormalities, anxiety-like behavior, and diminished locomotor activity [177], while B6.Nba2 mice show marked anxiety and mild depression [181]. Recent studies have documented several neuropsychiatric manifestations in PIL mice, including learning and memory disturbances [189] and reduced spontaneous activities [190]. These SLE mouse models can be utilized to investigate early molecular and cellular physiological changes leading to the occurrence of stroke and to explore the relationship between the progression of SLE and the risk factors directly related to stroke.

An alternative strategy involves exposing wild-type mice to chemical agents, offering a distinct approach to creating SLE models. This method mimics the influence of environmental factors on the development of lupus-specific manifestations. These models facilitate the exploration of non-genetic factors that trigger immune tolerance breakdown and provide a means to assess the effectiveness of therapeutic agents for SLE. Pristane, identified as hydrocarbon oil (2,6,10,14-tetramethylpentadecane), has the capacity to induce a diverse range of autoantibodies specific to or associated with SLE in mice (Table 4). The administration of pristane into the abdominal cavity of mice can induce various lupus-like symptoms, including ascitic fluid enriched with monoclonal antibodies and rheumatoid-like erosive arthritis [182]. However, despite the observation of most major clinical and laboratory manifestations of SLE, as defined by the American College of Rheumatology, in this pristane-induced lupus (PIL) model, there have been limited reports on NPSLE manifestations within this model [189,190,191]. 

This induced SLE model can serve as an excellent laboratory tool for investigating cardiovascular events, including the stroke risk associated with SLE. It may also contribute to understanding the impact of SLE severity on this association. Furthermore, this model enables the study of the molecular and cellular mechanisms involved in initiating stroke in SLE patients, which can help discover new markers and develop medications to manage the occurrence of stroke in these patients.

## 6. Management Gaps and Future Research Priorities for Discovering Medications to Treat Stroke in Systemic Lupus Erythematosus

The complexity of stroke pathogenesis in individuals with SLE poses a challenge to current management strategies. Valuable insights can be gained through a comprehensive analysis of each patient’s medical history, including disease progression, serum cholesterol levels, smoking habits, and antiphospholipid antibodies [28,192]. It is essential to conduct additional research to achieve a more profound understanding of the intricate relationship between stroke and SLE, particularly in patients with various risk factors or comorbidities. This research holds the potential to improve the management of stroke risk factors and occurrences in individuals with SLE. Medication combinations should be tailored to individual patients based on their symptoms and potential risk factors. Future research aimed at discovering new medications should consider these factors during developmental studies.

Attributing strokes to SLE remains challenging due to the influence of non-disease factors. Early stroke occurrence, particularly close to diagnosis, and a history of neuropsychiatric NPSLE manifestations may serve as predictors for future strokes [193]. Clinicians benefit from a validated attribution model that assists in identifying NP lupus manifestations, including CVAs [194]. The latest EULAR guidelines for SLE management now incorporate the concept of attribution, highlighting the importance of immunosuppressive therapy beyond thrombosis [160]. This can contribute to setting fundamental research directions by monitoring and managing the severity of SLE patient cases. It aims to understand the factors and mechanisms that contribute to the occurrence of stroke in association with SLE. This knowledge will enable the development of appropriate medications for individual patients, minimizing side effects.

When assessing cerebrovascular disease related to SLE, it is essential to conduct a thorough patient history and physical examination. The identification of causes based on symptoms, such as headaches indicating SAH, CVST, or vasculitis-related strokes, is crucial [195]. The sudden onset of focal neurological deficits may suggest an atherosclerotic or embolic origin [196]. Seizures, which are more frequent in vasculitis, may manifest across these conditions [197]. The examination should also encompass systemic signs of SLE activity, neurological assessments, and the use of the NIH Stroke Scale [79]. Based on these assessments, individualized medication plans should be developed for better management of each patient.

Unlike SLE, there are insufficient data regarding stroke in individuals with APS. Although some evidence suggests a connection between certain antibodies and stroke, further confirmatory studies are necessary. In contrast to the well-defined recommendations for stroke management in SLE, APS management lacks clear guidelines, resulting in uncertainties regarding optimal management, pathophysiology, and the role of non-criteria antiphospholipid antibodies. Therefore, it is necessary to develop improved management plans and identify effective medications to prevent stroke in APS patients.

While cerebrovascular complications in SLE are increasingly recognized, uncertainties persist regarding their prevalence, timing, and long-term outcomes, particularly with respect to acute strokes arising from various sources and mechanisms, such as intracerebral hemorrhage linked to vasculitis, hypertension, or aneurysm rupture. Evaluating SLE patients presenting with stroke-like symptoms necessitates the exclusion of secondary causes [13].

## 7. Concluding Remarks

SLE is an intractable, multisystemic, and relapsing disease primarily characterized by the loss of tolerance to self-antigens, immune complex formation, and diverse end-organ damages [198]. Nervous system involvement in SLE is a significant risk factor, impacting cognition, mood, and consciousness, known as neuropsychiatric lupus. This condition is linked to a poorer prognosis and increased cumulative organ damage [199,200]. The pathogenesis of NPSLE associated with strokes in SLE severity is believed to involve various pathogenic factors, including inflammatory mediators, autoantibodies, and immune cells, potentially disrupting the blood–brain barrier (BBB) and triggering an inflammatory process leading to glial activation, neurodegeneration, and behavioral deficits [201,202]. However, the mechanisms underlying NPSLE remain largely unknown, necessitating future studies to focus on understanding these various pathways and related complications.

While there has been substantial advancement in understanding SLE and APS, the diagnostic and therapeutic complexities posed by neurological manifestations persist for healthcare providers [140]. Consequently, additional basic and clinical research focused on unraveling the connection between APS and stroke, particularly the mechanisms by which antibodies trigger blood clot formation, could pave the way for the development of novel, validated diagnostic tools and effective management and medication strategies. These advancements have the potential to enhance control over the progressive pathophysiological nature of strokes associated with APS.

There is limited research on the use of antiplatelets or anticoagulants in SLE patients and their associations with ischemic and hemorrhagic strokes. Most prior studies have focused on the pathogenesis of strokes in SLE rather than medication usage. Beyond the utilization of anticoagulant medications in treating SLE, there is optimism for short- and long-term successes with the potential inclusion of several biologic drugs in standard lupus treatments. Anticipation is growing for a wider array of biologic drugs becoming available within the next five to ten years. This prospect offers hope for SLE patients in potentially averting the development of NPSLE, primarily due to the associated risk of nervous system disorders, particularly stroke.

The intricate pathophysiology of SLE presents a challenge, and one obvious and available tool is the use of primary lupus mouse models. However, these models may not be fully conducive to a deep understanding of SLE, possibly due to inherent differences in anatomy, immune function, and the varied expression of SLE between mice and humans. While no single mouse model can perfectly replicate human SLE, a range of these models has provided valuable insights into understanding the development of SLE. This may facilitate the initial experimental steps for further research aimed at comprehending the risk factors for various forms of stroke in SLE patients and exploring innovative treatment approaches, potentially including novel medication options.

Research aimed at understanding the occurrence of strokes in the context of SLE remains relatively limited. As efforts persist in addressing the complexities of replicating human SLE within mouse models, ongoing research endeavors are progressively enhancing our understanding of the molecular and cellular bases of this disease. This steadfast commitment has the potential to reveal novel treatment strategies based on new biological targets and medication approaches, ultimately advancing patient care.

## Figures and Tables

**Table 1:** Outlining studies and their key findings on stroke occurrence in SLE patients.

Type of Study	Findings	Reference
International inception cohort studies	The prevalence of stroke in SLE patients is 2.6% and 3.3%.	[18,19]
Retrospective cohort study	Patients with SLE who experience strokes suffer from extensive morbidity, elevated rates of premature mortality, and increased hospitalization costs.	[20]
Meta-analysis	Individuals with SLE face a 2-fold increased risk of ischemic stroke, a 3-fold elevated risk of intracerebral hemorrhage, and nearly a 4-fold increased risk of subarachnoid hemorrhage (SAH) compared to the general population, with the highest risk being even more pronounced in women and younger individuals (below 50 years of age), particularly within the first year following diagnosis.	[7]
Meta-analysis	SLE increases the risk of ischemic and hemorrhagic stroke, with the most pronounced impact in individuals under 50 years of age.	[21]
Two meta-analyses	The prevalence of cerebrovascular diseases in SLE patients ranges between 4.4% and 7.2%, although significant variability across studies was noted.	[21,22]
Cohort study	Ischemic strokes displayed a bimodal pattern, occurring either shortly after SLE diagnosis or after several years.	[23]
Population-based studies	Thrombotic events are most common in the initial five years of SLE, with the highest risks observed in the first year following SLE diagnosis.	[24,25]
Population-based studies	SLE significantly elevates the risk of stroke, particularly in patients younger than 50 years, leading to increased mortality and functional impairment when compared to non-SLE stroke patients.	[22,24,26,27]
Cohort study	Half of the hemorrhagic strokes occurred more than 10 years after the diagnosis of SLE.	[28]
Retrospective population-based cohort study	A severe lupus flare is strongly associated with an increased risk of ischemic and hemorrhagic strokes in SLE patients.	[29]
Retrospective cohort study	- Patients diagnosed with SLE had a higher incidence of stroke events (6.6%) in comparison to those without SLE where (3.8%) experienced strokes, even ten years after their initial SLE diagnosis. - The use of antiplatelet medications was associated with an elevated risk of hemorrhagic stroke in the SLE group.	[30]
Cohort study	An increased risk of stroke, myocardial infarction (MI), cardiovascular disease (CVD), and hypertension in patients with SLE compared to the general population, despite substantial variability among the included studies.	[31]

**Table 4:** Mouse models demonstrate neuropsychiatric symptoms in SLE.

Autoantibody	Findings	Reference
NZBWF1 mice	- The NZBWF1 mouse model, the leading SLE model, develops autoimmunity, anemia, proteinuria, and glomerulonephritis at 5 to 6 months. - NZB/W F1 and MRL/lpr mice exhibit neuropsychiatric symptoms but show delayed and inconsistent onset of SLE-related symptoms, progressing slowly, limiting their suitability for NPSLE research due to variability.	[174,175,176,177]
MRL/lpr mice	- MRL/lpr mice are genetically predisposed to spontaneous neuropsychiatric SLE. - NZB/W F1 and MRL/lpr mice exhibit neuropsychiatric symptoms but show delayed and inconsistent onset of SLE-related symptoms, progressing slowly, limiting their suitability for NPSLE research due to variability.	[178,179]
PIL mice	- PIL mice, inducible wild-type lupus models, precisely replicate clinical manifestations. - By week 2, PIL mice show increased brain cytokines and chemokines before behavioral deficits. - Cytokine dysregulation in PIL mice causes blood–brain barrier impairment, IgG deposition, neuronal damage, and glial activation, emphasizing the role of PIL mice in neuropsychiatric SLE research.	[180]
B6.Nba2 mice	- B6.Nba2 mice show notable anxiety and mild depression.	[181]
Wild-type mice + Pristane	- Pristane injection in mice induces lupus-like symptoms, including ascites with monoclonal antibodies and rheumatoid-like erosive arthritis. - Reports on neuropsychiatric SLE (NPSLE) manifestations in the pristane-induced lupus (PIL) model are limited, despite exhibiting major clinical and laboratory manifestations of SLE.	[182]

## Data Availability

Not applicable.

## References

[1] Cervera R., Khamashta M.A., Font J., Sebastiani G.D., Gil A., Lavilla P., Mejia J.C., Aydintug A.O., Chwalinska-Sadowska H., de Ramon E. (2003). Morbidity and mortality in systemic lupus erythematosus during a 10-year period: A comparison of early and late manifestations in a cohort of 1000 patients. Medicine.

[2] Unterman A., Nolte J.E., Boaz M., Abady M., Shoenfeld Y., Zandman-Goddard G. (2011). Neuropsychiatric syndromes in systemic lupus erythematosus: A meta-analysis. Semin. Arthritis Rheum..

[3] Saadatnia M., Sayed-Bonakdar Z., Mohammad-Sharifi G., Sarrami A.H. (2012). The necessity of stroke prevention in patients with systemic lupus erythematosus. J. Res. Med. Sci..

[4] Urowitz M.B., Gladman D., Ibanez D., Bae S.C., Sanchez-Guerrero J., Gordon C., Clarke A., Bernatsky S., Fortin P.R., Hanly J.G. (2010). Atherosclerotic vascular events in a multinational inception cohort of systemic lupus erythematosus. Arthritis Care Res..

[5] Mikdashi J., Handwerger B., Langenberg P., Miller M., Kittner S. (2007). Baseline disease activity, hyperlipidemia, and hypertension are predictive factors for ischemic stroke and stroke severity in systemic lupus erythematosus. Stroke.

[6] Chiu C.C., Huang C.C., Chan W.L., Chung C.M., Huang P.H., Lin S.J., Chen J.W., Leu H.B. (2012). Increased risk of ischemic stroke in patients with systemic lupus erythematosus: A nationwide population-based study. Intern. Med..

[7] Holmqvist M., Simard J.F., Asplund K., Arkema E.V. (2015). Stroke in systemic lupus erythematosus: A meta-analysis of population-based cohort studies. RMD Open.

[8] Saadatnia M., Sayed-Bonakdar Z., Mohammad-Sharifi G., Sarrami A.H. (2014). Prevalence and Prognosis of Cerebrovascular Accidents and its Subtypes Among Patients with Systemic Lupus Erythematosus in Isfahan, Iran: A Hospital Clinic-based Study. Int. J. Prev. Med..

[9] Govoni M., Bortoluzzi A., Padovan M., Silvagni E., Borrelli M., Donelli F., Ceruti S., Trotta F. (2016). The diagnosis and clinical management of the neuropsychiatric manifestations of lupus. J. Autoimmun..

[10] Sankowski R., Mader S., Valdes-Ferrer S.I. (2015). Systemic inflammation and the brain: Novel roles of genetic, molecular, and environmental cues as drivers of neurodegeneration. Front. Cell Neurosci..

[11] Ainiala H., Loukkola J., Peltola J., Korpela M., Hietaharju A. (2001). The prevalence of neuropsychiatric syndromes in systemic lupus erythematosus. Neurology.

[12] Fanouriakis A., Tziolos N., Bertsias G., Boumpas D.T. (2021). Update omicronn the diagnosis and management of systemic lupus erythematosus. Ann. Rheum. Dis..

[13] Nikolopoulos D., Fanouriakis A., Boumpas D.T. (2019). Cerebrovascular Events in Systemic Lupus Erythematosus: Diagnosis and Management. Mediterr. J. Rheumatol..

[14] Al-Ewaidat O.A., Naffaa M.M. (2024). Stroke risk in rheumatoid arthritis patients: Exploring connections and implications for patient care. Clin. Exp. Med..

[15] Shaban A., Leira E.C. (2019). Neurological Complications in Patients with Systemic Lupus Erythematosus. Curr. Neurol. Neurosci. Rep..

[16] Fernandez-Nebro A., Rua-Figueroa I., Lopez-Longo F.J., Galindo-Izquierdo M., Calvo-Alen J., Olive-Marques A., Ordonez-Canizares C., Martin-Martinez M.A., Blanco R., Melero-Gonzalez R. (2015). Cardiovascular Events in Systemic Lupus Erythematosus: A Nationwide Study in Spain From the RELESSER Registry. Medicine.

[17] Timlin H., Petri M. (2013). Transient ischemic attack and stroke in systemic lupus erythematosus. Lupus.

[18] Hanly J.G., Urowitz M.B., Su L., Sanchez-Guerrero J., Bae S.C., Gordon C., Wallace D.J., Isenberg D., Alarcon G.S., Merrill J.T. (2008). Short-term outcome of neuropsychiatric events in systemic lupus erythematosus upon enrollment into an international inception cohort study. Arthritis Rheum..

[19] Hanly J.G., Urowitz M.B., Su L., Bae S.C., Gordon C., Wallace D.J., Clarke A., Bernatsky S., Isenberg D., Rahman A. (2010). Prospective analysis of neuropsychiatric events in an international disease inception cohort of patients with systemic lupus erythematosus. Ann. Rheum. Dis..

[20] Wang I.K., Muo C.H., Chang Y.C., Liang C.C., Lin S.Y., Chang C.T., Yen T.H., Chuang F.R., Chen P.C., Huang C.C. (2012). Risks, subtypes, and hospitalization costs of stroke among patients with systemic lupus erythematosus: A retrospective cohort study in Taiwan. J. Rheumatol..

[21] Wiseman S.J., Ralston S.H., Wardlaw J.M. (2016). Cerebrovascular Disease in Rheumatic Diseases: A Systematic Review and Meta-Analysis. Stroke.

[22] Gu M.M., Wang X.P., Cheng Q.Y., Zhao Y.L., Zhang T.P., Li B.Z., Ye D.Q. (2019). A Meta-Analysis of Cardiovascular Events in Systemic Lupus Erythematosus. Immunol. Investig..

[23] Urowitz M.B., Gladman D.D., Anderson N.M., Su J., Romero-Diaz J., Bae S.C., Fortin P.R., Sanchez-Guerrero J., Clarke A., Bernatsky S. (2016). Cardiovascular events prior to or early after diagnosis of systemic lupus erythematosus in the systemic lupus international collaborating clinics cohort. Lupus Sci. Med..

[24] Arkema E.V., Svenungsson E., Von Euler M., Sjowall C., Simard J.F. (2017). Stroke in systemic lupus erythematosus: A Swedish population-based cohort study. Ann. Rheum. Dis..

[25] Avina-Zubieta J.A., To F., Vostretsova K., De Vera M., Sayre E.C., Esdaile J.M. (2017). Risk of Myocardial Infarction and Stroke in Newly Diagnosed Systemic Lupus Erythematosus: A General Population-Based Study. Arthritis Care Res..

[26] Hanly J.G., Li Q., Su L., Urowitz M.B., Gordon C., Bae S.C., Romero-Diaz J., Sanchez-Guerrero J., Bernatsky S., Clarke A.E. (2018). Cerebrovascular Events in Systemic Lupus Erythematosus: Results From an International Inception Cohort Study. Arthritis Care Res..

[27] Rossides M., Simard J.F., Svenungsson E., von Euler M., Arkema E.V. (2017). Mortality and Functionality after Stroke in Patients with Systemic Lupus Erythematosus. J. Rheumatol..

[28] Guraieb-Chahin P., Cantu-Brito C., Soto-Mota A., Guerrero-Torres L., Flores-Silva F., Chiquete E., Fragoso-Loyo H., Gonzalez-Duarte A., Valdes-Ferrer S.I. (2020). Stroke in systemic lupus erythematosus: Epidemiology, mechanism, and long-term outcome. Lupus.

[29] Chang K.C., Lin C.H., Chen P.L., Wu Y.H., Hou C.W., Huang J.A. (2023). Severe lupus flare is associated with a much higher risk of stroke among patients with SLE. Int. J. Stroke.

[30] Huang J.A., Lin C.H., Wu M.J., Chen Y.H., Chang K.C., Hou C.W. (2023). Ten-year follow-up investigation of stroke risk in systemic lupus erythematosus. Stroke Vasc. Neurol..

[31] Bello N., Meyers K.J., Workman J., Hartley L., McMahon M. (2023). Cardiovascular events and risk in patients with systemic lupus erythematosus: Systematic literature review and meta-analysis. Lupus.

[32] Liou T.H., Huang S.W., Lin J.W., Chang Y.S., Wu C.W., Lin H.W. (2014). Risk of stroke in patients with rheumatism: A nationwide longitudinal population-based study. Sci. Rep..

[33] Asanuma Y., Oeser A., Shintani A.K., Turner E., Olsen N., Fazio S., Linton M.F., Raggi P., Stein C.M. (2003). Premature coronary-artery atherosclerosis in systemic lupus erythematosus. N. Engl. J. Med..

[34] Blohorn A., Guegan-Massardier E., Triquenot A., Onnient Y., Tron F., Borg J.Y., Mihout B. (2002). Antiphospholipid antibodies in the acute phase of cerebral ischaemia in young adults: A descriptive study of 139 patients. Cerebrovasc. Dis..

[35] Vaudo G., Marchesi S., Gerli R., Allegrucci R., Giordano A., Siepi D., Pirro M., Shoenfeld Y., Schillaci G., Mannarino E. (2004). Endothelial dysfunction in young patients with rheumatoid arthritis and low disease activity. Ann. Rheum. Dis..

[36] Doria A., Shoenfeld Y., Wu R., Gambari P.F., Puato M., Ghirardello A., Gilburd B., Corbanese S., Patnaik M., Zampieri S. (2003). Risk factors for subclinical atherosclerosis in a prospective cohort of patients with systemic lupus erythematosus. Ann. Rheum. Dis..

[37] Romero-Diaz J., Garcia-Sosa I., Sanchez-Guerrero J. (2009). Thrombosis in systemic lupus erythematosus and other autoimmune diseases of recent onset. J. Rheumatol..

[38] Radic M., Martinovic Kaliterna D., Radic J. (2013). Vascular manifestations of systemic lupus erythematosis. Neth. J. Med..

[39] Candelario-Jalil E., Dijkhuizen R.M., Magnus T. (2022). Neuroinflammation, Stroke, Blood-Brain Barrier Dysfunction, and Imaging Modalities. Stroke.

[40] Jurcau A., Simion A. (2021). Neuroinflammation in Cerebral Ischemia and Ischemia/Reperfusion Injuries: From Pathophysiology to Therapeutic Strategies. Int. J. Mol. Sci..

[41] Wiseman S.J., Bastin M.E., Jardine C.L., Barclay G., Hamilton I.F., Sandeman E., Hunt D., Amft E.N., Thomson S., Belch J.F. (2016). Cerebral Small Vessel Disease Burden Is Increased in Systemic Lupus Erythematosus. Stroke.

[42] Cohen D., Rijnink E.C., Nabuurs R.J., Steup-Beekman G.M., Versluis M.J., Emmer B.J., Zandbergen M., van Buchem M.A., Allaart C.F., Wolterbeek R. (2017). Brain histopathology in patients with systemic lupus erythematosus: Identification of lesions associated with clinical neuropsychiatric lupus syndromes and the role of complement. Rheumatology.

[43] Esdaile J.M., Abrahamowicz M., Grodzicky T., Li Y., Panaritis C., du Berger R., Cote R., Grover S.A., Fortin P.R., Clarke A.E. (2001). Traditional Framingham risk factors fail to fully account for accelerated atherosclerosis in systemic lupus erythematosus. Arthritis Rheum..

[44] Gigante A., Barbano B., Liberatori M., Sardo L., Gasperini M.L., Rosato E., Cianci R., Amoroso A. (2013). Nephrotic syndrome and stroke. Int. J. Immunopathol. Pharmacol..

[45] Tsoi L.K., Mok C.C., Man B.L., Fu Y.P. (2021). Imaging Pattern and Outcome of Stroke in Patients With Systemic Lupus Erythematosus: A Case-control Study. J. Rheumatol..

[46] Parker K., Ragy O., Hamilton P., Thachil J., Kanigicherla D. (2023). Thromboembolism in nephrotic syndrome: Controversies and uncertainties. Res. Pract. Thromb. Haemost..

[47] Huang W.Y., Chang C.W., Chen K.H., Chang C.H., Wu H.C., Chang K.H. (2023). Characteristics of acute ischemic stroke in patients with Nephrotic syndrome. Ren. Fail..

[48] Roy C., Deschaintre Y., Sabbagh R., Roy D., Cardinal H., Bollee G. (2017). Ischemic Stroke of Possible Embolic Etiology Associated With Nephrotic Syndrome. Kidney Int. Rep..

[49] Wu D., Han M., Lee S. (2023). Risk of acute stroke in young patients with new-onset lupus nephritis: A case series (P9-5.028). Neurol. J..

[50] Lee M.A., Hwang B.W., Lee D.K., Lee C.J., Kim J.H., Ahn S.H. (2022). Acute Stroke Caused by Large Vessel Vasculitis in a Patient with Systemic Lupus Erythematosus. J. Neurosonol. Neuroimaging.

[51] Bustamante J.G., Goyal A., Singhal M. (2023). Antiphospholipid Syndrome.

[52] Miyakis S., Lockshin M.D., Atsumi T., Branch D.W., Brey R.L., Cervera R., Derksen R.H., PG D.E.G., Koike T., Meroni P.L. (2006). International consensus statement on an update of the classification criteria for definite antiphospholipid syndrome (APS). J. Thromb. Haemost..

[53] Pasoto S.G., Adriano de Oliveira Martins V., Bonfa E. (2019). Sjogren’s syndrome and systemic lupus erythematosus: Links and risks. Open Access Rheumatol..

[54] Rodriguez M.F., Asnal C., Gobbi C.A., Pellet A.C.C., Herscovich N., Amitrano C., Demarchi J., Noe D.D., Segura C., Caeiro F. (2022). Primary Sjogren syndrome and development of another autoimmune rheumatic disease during the follow-up. Adv. Rheumatol..

[55] Sammaritano L.R. (2020). Antiphospholipid syndrome. Best Pract. Res. Clin. Rheumatol..

[56] El Hasbani G., Saliba A.N., Uthman I., Taher A.T. (2023). Hematological manifestations of antiphospholipid syndrome: Going beyond thrombosis. Blood Rev..

[57] Mok C.C., Ho L.Y., To C.H. (2009). Annual incidence and standardized incidence ratio of cerebrovascular accidents in patients with systemic lupus erythematosus. Scand. J. Rheumatol..

[58] Fleetwood T., Cantello R., Comi C. (2018). Antiphospholipid Syndrome and the Neurologist: From Pathogenesis to Therapy. Front. Neurol..

[59] Gao N., Wang Z.L., Li M.T., Han S.M., Dang Y.Q., Zhang F.C., Shi T.Y., Zhang L.N., Zeng X.F. (2013). Clinical characteristics and risk factors of intracranial hemorrhage in systemic lupus erythematosus. Lupus.

[60] Torne R., Rodriguez-Hernandez A., Bernard T., Arikan Abello F., Vilalta Castan J., Sahuquillo J. (2015). Subarachnoid hemorrhage in systemic lupus erythematosus: Systematic review and report of three cases. Clin. Neurol. Neurosurg..

[61] Tsukamoto E., Tanei T., Senda J., Kato T., Naito T., Ishii K., Okada K., Hasegawa T. (2020). Subarachnoid Hemorrhage After Ischemic Stroke Associated with Systemic Lupus Erythematosus and Antiphospholipid Syndrome. World Neurosurg..

[62] de Amorim L.C., Maia F.M., Rodrigues C.E. (2017). Stroke in systemic lupus erythematosus and antiphospholipid syndrome: Risk factors, clinical manifestations, neuroimaging, and treatment. Lupus.

[63] Valdes-Ferrer S.I., Vega F., Cantu-Brito C., Ceballos-Ceballos J., Estanol B., Garcia-Ramos G., Cabral A.R. (2008). Cerebral changes in SLE with or without antiphospholipid syndrome. a case-control MRI study. J. Neuroimaging.

[64] Flores-Silva F.D., Longoria-Lozano O., Aguirre-Villarreal D., Senties-Madrid H., Vega-Boada F., Diaz de Leon-Sanchez E., Murra-Anton S., Morales-Moreno S., Quintanilla-Gonzalez L., Fragoso-Loyo H. (2018). Natural history of longitudinally extensive transverse myelitis in 35 Hispanic patients with systemic lupus erythematosus: Good short-term functional outcome and paradoxical increase in long-term mortality. Lupus.

[65] Ricarte I.F., Dutra L.A., Barsottini O.G.P., Souza A.W.S., Andrade D.C.O., Mangueira C., Silva G.S. (2019). Transcranial Doppler findings in antiphospholipid syndrome. Lupus.

[66] Alarcon-Segovia D., Cardiel M.H., Reyes E. (1989). Antiphospholipid arterial vasculopathy. J. Rheumatol..

[67] Hughson M.D., McCarty G.A., Brumback R.A. (1995). Spectrum of vascular pathology affecting patients with the antiphospholipid syndrome. Hum. Pathol..

[68] Leung W.H., Wong K.L., Lau C.P., Wong C.K., Liu H.W. (1990). Association between antiphospholipid antibodies and cardiac abnormalities in patients with systemic lupus erythematosus. Am. J. Med..

[69] Zuily S., Regnault V., Selton-Suty C., Eschwege V., Bruntz J.F., Bode-Dotto E., De Maistre E., Dotto P., Perret-Guillaume C., Lecompte T. (2011). Increased risk for heart valve disease associated with antiphospholipid antibodies in patients with systemic lupus erythematosus: Meta-analysis of echocardiographic studies. Circulation.

[70] Hughes G.R. (2003). Migraine, memory loss, and “multiple sclerosis”. Neurological features of the antiphospholipid (Hughes’) syndrome. Postgrad. Med. J..

[71] Sciascia S., Radin M., Cecchi I., Rubini E., Scotta A., Rolla R., Montaruli B., Pergolini P., Mengozzi G., Muccini E. (2019). Reliability of Lupus Anticoagulant and Anti-phosphatidylserine/prothrombin Autoantibodies in Antiphospholipid Syndrome: A Multicenter Study. Front. Immunol..

[72] de Souza A.W., Silva N.P., de Carvalho J.F., D’Almeida V., Noguti M.A., Sato E.I. (2007). Impact of hypertension and hyperhomocysteinemia on arterial thrombosis in primary antiphospholipid syndrome. Lupus.

[73] Sciascia S., Sanna G., Khamashta M.A., Cuadrado M.J., Erkan D., Andreoli L., Bertolaccini M.L., Action A.P.S. (2015). The estimated frequency of antiphospholipid antibodies in young adults with cerebrovascular events: A systematic review. Ann. Rheum. Dis..

[74] Petri M., Rheinschmidt M., Whiting-O’Keefe Q., Hellmann D., Corash L. (1987). The frequency of lupus anticoagulant in systemic lupus erythematosus. A study of sixty consecutive patients by activated partial thromboplastin time, Russell viper venom time, and anticardiolipin antibody level. Ann. Intern. Med..

[75] Hanly J.G., Urowitz M.B., Su L., Bae S.C., Gordon C., Clarke A., Bernatsky S., Vasudevan A., Isenberg D., Rahman A. (2011). Autoantibodies as biomarkers for the prediction of neuropsychiatric events in systemic lupus erythematosus. Ann. Rheum. Dis..

[76] Sanna G., D’Cruz D., Cuadrado M.J. (2006). Cerebral manifestations in the antiphospholipid (Hughes) syndrome. Rheum. Dis. Clin. N. Am..

[77] Oosting J.D., Derksen R.H., Blokzijl L., Sixma J.J., de Groot P.G. (1992). Antiphospholipid antibody positive sera enhance endothelial cell procoagulant activity—Studies in a thrombosis model. Thromb. Haemost..

[78] Meroni P.L., Tincani A., Sepp N., Raschi E., Testoni C., Corsini E., Cavazzana I., Pellegrini S., Salmaggi A. (2003). Endothelium and the brain in CNS lupus. Lupus.

[79] Connor P., Hunt B.J. (2003). Cerebral haemostasis and antiphospholipid antibodies. Lupus.

[80] Zolyan A., Crawford J.R. (2022). Brainstem stroke in a patient with systemic lupus erythematosus and triple antiphospholipid antibody profile. BMJ Case Rep..

[81] Khangura R.K., Cooper S., Luo G.-Y. (2019). Antiphospholipid Antibody Syndrome:Pathogenesis, Diagnosis, and Managementin Pregnancy. Matern.-Fetal Med..

[82] Chayoua W., Kelchtermans H., Moore G.W., Musial J., Wahl D., de Laat B., Devreese K.M.J. (2018). Identification of high thrombotic risk triple-positive antiphospholipid syndrome patients is dependent on anti-cardiolipin and anti-β_2_glycoprotein I antibody detection assays. J. Thromb. Haemost..

[83] Panichpisal K., Rozner E., Levine S.R. (2012). The management of stroke in antiphospholipid syndrome. Curr. Rheumatol. Rep..

[84] Pengo V., Denas G., Zoppellaro G., Jose S.P., Hoxha A., Ruffatti A., Andreoli L., Tincani A., Cenci C., Prisco D. (2018). Rivaroxaban vs warfarin in high-risk patients with antiphospholipid syndrome. Blood.

[85] Wigren M., Svenungsson E., Mattisson I.Y., Gustafsson J.T., Gunnarsson I., Zickert A., Elvin K., Jensen-Urstad K., Bengtsson A., Gullstrand B. (2018). Cardiovascular disease in systemic lupus erythematosus is associated with increased levels of biomarkers reflecting receptor-activated apoptosis. Atherosclerosis.

[86] Piga M., Arnaud L. (2021). The Main Challenges in Systemic Lupus Erythematosus: Where Do We Stand?. J. Clin. Med..

[87] McMahon M., Skaggs B., Grossman J., Wong W.K., Sahakian L., Chen W., Hahn B. (2019). Comparison of PREDICTS atherosclerosis biomarker changes after initiation of new treatments in patients with SLE. Lupus Sci. Med..

[88] Skaggs B.J., Grossman J., Sahakian L., Perry L., FitzGerald J., Charles-Schoeman C., Gorn A., Taylor M., Moriarty J., Ragavendra N. (2021). A Panel of Biomarkers Associates With Increased Risk for Cardiovascular Events in Women With Systemic Lupus Erythematosus. ACR Open Rheumatol..

[89] McMahon M., Skaggs B. (2014). Pathogenesis and treatment of atherosclerosis in lupus. Rheum. Dis. Clin. N. Am..

[90] Blachut D., Przywara-Chowaniec B., Tomasik A., Kukulski T., Morawiec B. (2023). Update of Potential Biomarkers in Risk Prediction and Monitoring of Atherosclerosis in Systemic Lupus Erythematosus to Prevent Cardiovascular Disease. Biomedicines.

[91] Aringer M., Costenbader K., Daikh D., Brinks R., Mosca M., Ramsey-Goldman R., Smolen J.S., Wofsy D., Boumpas D.T., Kamen D.L. (2019). 2019 European League Against Rheumatism/American College of Rheumatology Classification Criteria for Systemic Lupus Erythematosus. Arthritis Rheumatol..

[92] de Groot P.G., de Laat B. (2017). Mechanisms of thrombosis in systemic lupus erythematosus and antiphospholipid syndrome. Best Pract. Res. Clin. Rheumatol..

[93] Sun Y.A., Han Q., Hou X.H., Peng X.Z., Tong L., Zheng X., Yu J.T., Tan L. (2020). Association of antinuclear antibodies with the risk of intracranial arterial stenosis. Aging.

[94] Solow E.B., Vongpatanasin W., Skaug B., Karp D.R., Ayers C., de Lemos J.A. (2018). Antinuclear antibodies in the general population: Positive association with inflammatory and vascular biomarkers but not traditional cardiovascular risk factors. Clin. Exp. Rheumatol..

[95] Henriquez S., Legris N., Chretien P., Hacein-Bey-Abina S., Henry J., Denier C., Noel N. (2021). Discovery of Anti-SS-A Antibodies during Stroke Investigations in Young Adults: What Impact?. J. Stroke Cerebrovasc. Dis..

[96] Yang F., Yang Y., Zeng W. (2021). Blockade of anti-dsDNA ameliorates systemic lupus erythematosus in MRL/Faslpr mice through ameliorating inflammation via the PKCdelta-NLRC4 axis. Biochem. Cell Biol..

[97] Pisetsky D.S., Lipsky P.E. (2020). New insights into the role of antinuclear antibodies in systemic lupus erythematosus. Nat. Rev. Rheumatol..

[98] Kushner M.J. (1990). Prospective study of anticardiolipin antibodies in stroke. Stroke.

[99] Chakravarty K.K., Byron M.A., Webley M., Durkin C.J., al-Hillawi A.H., Bodley R., Wozniak J. (1991). Antibodies to cardiolipin in stroke: Association with mortality and functional recovery in patients without systemic lupus erythematosus. Q. J. Med..

[100] Ginsburg K.S., Liang M.H., Newcomer L., Goldhaber S.Z., Schur P.H., Hennekens C.H., Stampfer M.J. (1992). Anticardiolipin antibodies and the risk for ischemic stroke and venous thrombosis. Ann. Intern. Med..

[101] Hess D.C., Krauss J., Adams R.J., Nichols F.T., Zhang D., Rountree H.A. (1991). Anticardiolipin antibodies: A study of frequency in TIA and stroke. Neurology.

[102] de Mast Q., Molhoek J.E., van der Ven A.J., Gray W.K., de Groot P.G., Jusabani A., Mugusi F., Urbanus R.T., Walker R.W. (2016). Antiphospholipid Antibodies and the Risk of Stroke in Urban and Rural Tanzania: A Community-Based Case-Control Study. Stroke.

[103] Tanne D., D’Olhaberriague L., Schultz L.R., Salowich-Palm L., Sawaya K.L., Levine S.R. (1999). Anticardiolipin antibodies and their associations with cerebrovascular risk factors. Neurology.

[104] Lanthier S., Kirkham F.J., Mitchell L.G., Laxer R.M., Atenafu E., Male C., Prengler M., Domi T., Chan A.K., Liesner R. (2004). Increased anticardiolipin antibody IgG titers do not predict recurrent stroke or TIA in children. Neurology.

[105] Bala M.M., Celinska-Lowenhoff M., Szot W., Padjas A., Kaczmarczyk M., Swierz M.J., Undas A. (2020). Antiplatelet and anticoagulant agents for secondary prevention of stroke and other thromboembolic events in people with antiphospholipid syndrome. Cochrane Database Syst. Rev..

[106] Drosos G.C., Vedder D., Houben E., Boekel L., Atzeni F., Badreh S., Boumpas D.T., Brodin N., Bruce I.N., Gonzalez-Gay M.A. (2022). EULAR recommendations for cardiovascular risk management in rheumatic and musculoskeletal diseases, including systemic lupus erythematosus and antiphospholipid syndrome. Ann. Rheum. Dis..

[107] Urbanus R.T., Siegerink B., Roest M., Rosendaal F.R., de Groot P.G., Algra A. (2009). Antiphospholipid antibodies and risk of myocardial infarction and ischaemic stroke in young women in the RATIO study: A case-control study. Lancet Neurol..

[108] Brey R.L., Abbott R.D., Curb J.D., Sharp D.S., Ross G.W., Stallworth C.L., Kittner S.J. (2001). beta(2)-Glycoprotein 1-dependent anticardiolipin antibodies and risk of ischemic stroke and myocardial infarction: The honolulu heart program. Stroke.

[109] de Groot P.G., Urbanus R.T. (2012). The significance of autoantibodies against β_2_-glycoprotein I. Blood.

[110] Amory C.F., Levine S.R., Brey R.L., Gebregziabher M., Tuhrim S., Tilley B.C., Simpson A.C., Sacco R.L., Mohr J.P., Collaborators A.-W. (2015). Antiphospholipid Antibodies and Recurrent Thrombotic Events: Persistence and Portfolio. Cerebrovasc. Dis..

[111] Forastiero R., Martinuzzo M., Pombo G., Puente D., Rossi A., Celebrin L., Bonaccorso S., Aversa L. (2005). A prospective study of antibodies to β_2_-glycoprotein I and prothrombin, and risk of thrombosis. J. Thromb. Haemost..

[112] Liu T., Gu J., Wan L., Hu Q., Teng J., Liu H., Cheng X., Ye J., Su Y., Sun Y. (2020). “Non-criteria” antiphospholipid antibodies add value to antiphospholipid syndrome diagnoses in a large Chinese cohort. Arthritis Res. Ther..

[113] Cojocaru I.M., Cojocaru M., Musuroi C., Botezat M. (2003). Study of anti-cardiolipin and anti-β_2_-glycoprotein I antibodies in patients with ischemic stroke. Rom. J. Intern. Med..

[114] Saidi S., Mahjoub T., Almawi W.Y. (2009). Lupus anticoagulants and anti-phospholipid antibodies as risk factors for a first episode of ischemic stroke. J. Thromb. Haemost..

[115] Galli M., Luciani D., Bertolini G., Barbui T. (2003). Lupus anticoagulants are stronger risk factors for thrombosis than anticardiolipin antibodies in the antiphospholipid syndrome: A systematic review of the literature. Blood.

[116] Ioannidis S., Mavridis M., Mitsias P.D. (2018). Ischemic stroke as initial manifestation of systemic lupus erythematosus: A case report and review of the literature. eNeurologicalSci.

[117] Urbanski G., Yelnik C.M., Maillard H., Launay D., Dubucquoi S., Hachulla E., Hatron P.Y., Lambert M. (2018). Antiphospholipid Syndrome With Isolated Isotype M Anticardiolipin and/or Anti-B2GPI Antibody Is Associated With Stroke. Stroke.

[118] Sharma S., Tyagi T., Antoniak S. (2022). Platelet in thrombo-inflammation: Unraveling new therapeutic targets. Front. Immunol..

[119] Alvis-Miranda H.R., Milena Castellar-Leones S., Alcala-Cerra G., Rafael Moscote-Salazar L. (2013). Cerebral sinus venous thrombosis. J. Neurosci. Rural Pract..

[120] Shen J., Tao Z., Chen W., Sun J., Li Y., Fu F. (2022). Malignant Isolated Cortical Vein Thrombosis as the Initial Manifestation of Primary Antiphospholipid Syndrome: Lessons on Diagnosis and Management From a Case Report. Front. Immunol..

[121] Hu C., Li S., Xie Z., You H., Jiang H., Shi Y., Qi W., Zhao J., Wang Q., Tian X. (2021). Evaluation of the Diagnostic Value of Non-criteria Antibodies for Antiphospholipid Syndrome Patients in a Chinese Cohort. Front. Immunol..

[122] Naranjo L., Ostos F., Gil-Etayo F.J., Hernandez-Gallego J., Cabrera-Marante O., Pleguezuelo D.E., Diaz-Simon R., Cerro M., Lora D., Martinez-Salio A. (2021). Presence of Extra-Criteria Antiphospholipid Antibodies Is an Independent Risk Factor for Ischemic Stroke. Front. Cardiovasc. Med..

[123] Kahles T., Humpich M., Steinmetz H., Sitzer M., Lindhoff-Last E. (2005). Phosphatidylserine IgG and beta-2-glycoprotein I IgA antibodies may be a risk factor for ischaemic stroke. Rheumatology.

[124] Staub H.L., Norman G.L., Crowther T., da Cunha V.R., Polanczyk A., Bohn J.M., Fernandes J.G., Chahade W.H., von Muhlen C.A. (2003). Antibodies to the atherosclerotic plaque components β_2_-glycoprotein I and heat-shock proteins as risk factors for acute cerebral ischemia. Arq. Neuropsiquiatr..

[125] Mullen M.T., Messe S.R., Kasner S.E., Sansing L., Husain M.R., Norman G.L., Shums Z., Cucchiara B.L. (2012). Anti-Phosphatidylserine-Prothrombin Antibodies are Associated with Outcome in a TIA Cohort. Front. Neurol..

[126] Kim Y., Kim S.Y. (2020). Antiphospholipid Antibody and Recurrent Ischemic Stroke: A Systematic Review and Meta-Analysis. Stroke.

[127] Brouwer J.L., Bijl M., Veeger N.J., Kluin-Nelemans H.C., van der Meer J. (2004). The contribution of inherited and acquired thrombophilic defects, alone or combined with antiphospholipid antibodies, to venous and arterial thromboembolism in patients with systemic lupus erythematosus. Blood.

[128] Soare A.M., Popa C. (2010). Deficiencies of proteins C, S and antithrombin and activated protein C resistance–their involvement in the occurrence of Arterial thromboses. J. Med. Life.

[129] Simioni P. (2006). Who should be tested for thrombophilia?. Curr. Opin. Hematol..

[130] Chiasakul T., De Jesus E., Tong J., Chen Y., Crowther M., Garcia D., Chai-Adisaksopha C., Messe S.R., Cuker A. (2019). Inherited Thrombophilia and the Risk of Arterial Ischemic Stroke: A Systematic Review and Meta-Analysis. J. Am. Heart Assoc..

[131] Choi M.Y., Guan H., Yoshida K., Paudel M., Kargere B.A., Li D., Ellrodt J., Stevens E., Cai T., Weber B.N. (2024). Personalizing cardiovascular risk prediction for patients with systemic lupus erythematosus. Semin. Arthritis Rheum..

[132] McKeon K.P., Jiang S.H. (2020). Treatment of systemic lupus erythematosus. Aust. Prescr..

[133] Tektonidou M.G., Wang Z., Dasgupta A., Ward M.M. (2015). Burden of Serious Infections in Adults With Systemic Lupus Erythematosus: A National Population-Based Study, 1996–2011. Arthritis Care Res..

[134] Merrell M., Shulman L.E. (1955). Determination of prognosis in chronic disease, illustrated by systemic lupus erythematosus. J. Chronic Dis..

[135] Bernatsky S., Boivin J.F., Joseph L., Manzi S., Ginzler E., Gladman D.D., Urowitz M., Fortin P.R., Petri M., Barr S. (2006). Mortality in systemic lupus erythematosus. Arthritis Rheum..

[136] Borchers A.T., Keen C.L., Shoenfeld Y., Gershwin M.E. (2004). Surviving the butterfly and the wolf: Mortality trends in systemic lupus erythematosus. Autoimmun. Rev..

[137] Frodlund M., Reid S., Wettero J., Dahlstrom O., Sjowall C., Leonard D. (2019). The majority of Swedish systemic lupus erythematosus patients are still affected by irreversible organ impairment: Factors related to damage accrual in two regional cohorts. Lupus.

[138] Gladman D.D., Urowitz M.B., Rahman P., Ibanez D., Tam L.S. (2003). Accrual of organ damage over time in patients with systemic lupus erythematosus. J. Rheumatol..

[139] Rodrigues C.E., Carvalho J.F., Shoenfeld Y. (2010). Neurological manifestations of antiphospholipid syndrome. Eur. J. Clin. Investig..

[140] Bertsias G.K., Ioannidis J.P., Aringer M., Bollen E., Bombardieri S., Bruce I.N., Cervera R., Dalakas M., Doria A., Hanly J.G. (2010). EULAR recommendations for the management of systemic lupus erythematosus with neuropsychiatric manifestations: Report of a task force of the EULAR standing committee for clinical affairs. Ann. Rheum. Dis..

[141] Gaziano J.M., Brotons C., Coppolecchia R., Cricelli C., Darius H., Gorelick P.B., Howard G., Pearson T.A., Rothwell P.M., Ruilope L.M. (2018). Use of aspirin to reduce risk of initial vascular events in patients at moderate risk of cardiovascular disease (ARRIVE): A randomised, double-blind, placebo-controlled trial. Lancet.

[142] Petri M. (1996). Hydroxychloroquine use in the Baltimore Lupus Cohort: Effects on lipids, glucose and thrombosis. Lupus.

[143] Ruiz-Irastorza G., Egurbide M.V., Pijoan J.I., Garmendia M., Villar I., Martinez-Berriotxoa A., Erdozain J.G., Aguirre C. (2006). Effect of antimalarials on thrombosis and survival in patients with systemic lupus erythematosus. Lupus.

[144] Tektonidou M.G., Laskari K., Panagiotakos D.B., Moutsopoulos H.M. (2009). Risk factors for thrombosis and primary thrombosis prevention in patients with systemic lupus erythematosus with or without antiphospholipid antibodies. Arthritis Rheum..

[145] Bertsias G., Ioannidis J.P., Boletis J., Bombardieri S., Cervera R., Dostal C., Font J., Gilboe I.M., Houssiau F., Huizinga T. (2008). EULAR recommendations for the management of systemic lupus erythematosus. Report of a Task Force of the EULAR Standing Committee for International Clinical Studies Including Therapeutics. Ann. Rheum. Dis..

[146] Khamashta M.A., Cuadrado M.J., Mujic F., Taub N.A., Hunt B.J., Hughes G.R. (1995). The management of thrombosis in the antiphospholipid-antibody syndrome. N. Engl. J. Med..

[147] Ruiz-Irastorza G., Hunt B.J., Khamashta M.A. (2007). A systematic review of secondary thromboprophylaxis in patients with antiphospholipid antibodies. Arthritis Rheum..

[148] Johnson S.G., Rogers K., Delate T., Witt D.M. (2008). Outcomes associated with combined antiplatelet and anticoagulant therapy. Chest.

[149] Tektonidou M.G., Andreoli L., Limper M., Amoura Z., Cervera R., Costedoat-Chalumeau N., Cuadrado M.J., Dorner T., Ferrer-Oliveras R., Hambly K. (2019). EULAR recommendations for the management of antiphospholipid syndrome in adults. Ann. Rheum. Dis..

[150] Cohen H., Hunt B.J., Efthymiou M., Arachchillage D.R., Mackie I.J., Clawson S., Sylvestre Y., Machin S.J., Bertolaccini M.L., Ruiz-Castellano M. (2016). Rivaroxaban versus warfarin to treat patients with thrombotic antiphospholipid syndrome, with or without systemic lupus erythematosus (RAPS): A randomised, controlled, open-label, phase 2/3, non-inferiority trial. Lancet Haematol..

[151] Fanouriakis A., Pamfil C., Rednic S., Sidiropoulos P., Bertsias G., Boumpas D.T. (2016). Is it primary neuropsychiatric systemic lupus erythematosus? Performance of existing attribution models using physician judgment as the gold standard. Clin. Exp. Rheumatol..

[152] Schiavi C., Marri L., Negrini S. (2023). Arterial thrombosis triggered by methotrexate-induced hyperhomocysteinemia in a systemic lupus erythematosus patient with antiphospholipid antibodies. Thromb. J..

[153] Aguiar R., Araujo C., Martins-Coelho G., Isenberg D. (2017). Use of Rituximab in Systemic Lupus Erythematosus: A Single Center Experience Over 14 Years. Arthritis Care Res..

[154] Hui-Yuen J.S., Reddy A., Taylor J., Li X., Eichenfield A.H., Bermudez L.M., Starr A.J., Imundo L.F., Buyon J., Furie R.A. (2015). Safety and Efficacy of Belimumab to Treat Systemic Lupus Erythematosus in Academic Clinical Practices. J. Rheumatol..

[155] Tummala R., Abreu G., Pineda L., Michaels M.A., Kalyani R.N., Furie R.A., Morand E.F. (2021). Safety profile of anifrolumab in patients with active SLE: An integrated analysis of phase II and III trials. Lupus Sci. Med..

[156] Schreiber K., Sciascia S., de Groot P.G., Devreese K., Jacobsen S., Ruiz-Irastorza G., Salmon J.E., Shoenfeld Y., Shovman O., Hunt B.J. (2018). Antiphospholipid syndrome. Nat. Rev. Dis. Primers.

[157] Yacoub M.S., Khine J., Najar A., Yadlapalli S. (2022). Evidence of Thrombogenesis Recurrence Induced by IgA Antiphospholipid Antibody β_2_ Glycoprotein I-Dependent in Early Adulthood. Cureus.

[158] Frances C., Papo T., Wechsler B., Laporte J.L., Biousse V., Piette J.C. (1999). Sneddon syndrome with or without antiphospholipid antibodies. A comparative study in 46 patients. Medicine.

[159] Garcia-Rodriguez L.A., Gaist D., Morton J., Cookson C., Gonzalez-Perez A. (2013). Antithrombotic drugs and risk of hemorrhagic stroke in the general population. Neurology.

[160] Fanouriakis A., Kostopoulou M., Alunno A., Aringer M., Bajema I., Boletis J.N., Cervera R., Doria A., Gordon C., Govoni M. (2019). 2019 update of the EULAR recommendations for the management of systemic lupus erythematosus. Ann. Rheum. Dis..

[161] Burmester G.R., Bijlsma J.W.J., Cutolo M., McInnes I.B. (2017). Managing rheumatic and musculoskeletal diseases-past, present and future. Nat. Rev. Rheumatol..

[162] Lee D.S.W., Rojas O.L., Gommerman J.L. (2021). B cell depletion therapies in autoimmune disease: Advances and mechanistic insights. Nat. Rev. Drug Discov..

[163] Fanouriakis A., Adamichou C., Koutsoviti S., Panopoulos S., Staveri C., Klagou A., Tsalapaki C., Pantazi L., Konsta S., Mavragani C.P. (2018). Low disease activity-irrespective of serologic status at baseline-associated with reduction of corticosteroid dose and number of flares in patients with systemic lupus erythematosus treated with belimumab: A real-life observational study. Semin. Arthritis Rheum..

[164] Agnihotri P., Fazel-Rezai R., Kaabouch N. Comparative analysis of various brain imaging techniques. Proceedings of the 2010 Annual International Conference of the IEEE Engineering in Medicine and Biology.

[165] Venturelli V., Isenberg D.A. (2023). Targeted Therapy for SLE-What Works, What Doesn’t, What’s Next. J. Clin. Med..

[166] Md Yusof M.Y., Shaw D., El-Sherbiny Y.M., Dunn E., Rawstron A.C., Emery P., Vital E.M. (2017). Predicting and managing primary and secondary non-response to rituximab using B-cell biomarkers in systemic lupus erythematosus. Ann. Rheum. Dis..

[167] Collins C.E., Cortes-Hernandez J., Garcia M.A., von Kempis J., Schwarting A., Touma Z., Kurtinecz M., Gairy K. (2020). Real-World Effectiveness of Belimumab in the Treatment of Systemic Lupus Erythematosus: Pooled Analysis of Multi-Country Data from the OBSErve Studies. Rheumatol. Ther..

[168] Freitas S., Mozo Ruiz M., Costa Carneiro A., Isenberg D.A. (2020). Why do some patients with systemic lupus erythematosus fail to respond to B-cell depletion using rituximab?. Clin. Exp. Rheumatol..

[169] Hennessey A., Lukawska J., Cambridge G., Isenberg D., Leandro M. (2019). Adverse infusion reactions to rituximab in systemic lupus erythematosus: A retrospective analysis. BMC Rheumatol..

[170] Katarzyna P.B., Wiktor S., Ewa D., Piotr L. (2023). Current treatment of systemic lupus erythematosus: A clinician’s perspective. Rheumatol. Int..

[171] Stojan G., Petri M. (2017). The risk benefit ratio of glucocorticoids in SLE: Have things changed over the past 40 years?. Curr. Treat. Opt. Rheumatol..

[172] Li W., Titov A.A., Morel L. (2017). An update on lupus animal models. Curr. Opin. Rheumatol..

[173] Richard M.L., Gilkeson G. (2018). Mouse models of lupus: What they tell us and what they don’t. Lupus Sci. Med..

[174] Perry D., Sang A., Yin Y., Zheng Y.Y., Morel L. (2011). Murine models of systemic lupus erythematosus. J. Biomed. Biotechnol..

[175] Rottman J.B., Willis C.R. (2010). Mouse models of systemic lupus erythematosus reveal a complex pathogenesis. Vet. Pathol..

[176] Monneaux F., Dumortier H., Steiner G., Briand J.P., Muller S. (2001). Murine models of systemic lupus erythematosus: B and T cell responses to spliceosomal ribonucleoproteins in MRL/Fas(lpr) and (NZB x NZW)F(1) lupus mice. Int. Immunol..

[177] Dubois E.L., Horowitz R.E., Demopoulos H.B., Teplitz R. (1966). NZB/NZW mice as a model of systemic lupus erythematosus. JAMA.

[178] Gulinello M., Putterman C. (2011). The MRL/lpr mouse strain as a model for neuropsychiatric systemic lupus erythematosus. J. Biomed. Biotechnol..

[179] Ballok D.A. (2007). Neuroimmunopathology in a murine model of neuropsychiatric lupus. Brain Res. Rev..

[180] Yun Y., Wang X., Xu J., Jin C., Chen J., Wang X., Wang J., Qin L., Yang P. (2023). Pristane induced lupus mice as a model for neuropsychiatric lupus (NPSLE). Behav. Brain Funct..

[181] Browne K., Zhang E., Sullivan J.K., Evonuk K.S., DeSilva T.M., Jorgensen T.N. (2021). Lupus-prone B6.Nba2 male and female mice display anti-DWEYS reactivity and a neuropsychiatric phenotype. Brain Behav. Immun..

[182] Zeggar S., Watanabe K.S., Teshigawara S., Hiramatsu S., Katsuyama T., Katsuyama E., Watanabe H., Matsumoto Y., Kawabata T., Sada K.E. (2018). Role of Lgals9 Deficiency in Attenuating Nephritis and Arthritis in BALB/c Mice in a Pristane-Induced Lupus Model. Arthritis Rheumatol..

[183] Vo A., Volpe B.T., Tang C.C., Schiffer W.K., Kowal C., Huerta P.T., Ulug A.M., Dewey S.L., Eidelberg D., Diamond B. (2014). Regional brain metabolism in a murine systemic lupus erythematosus model. J. Cereb. Blood Flow. Metab..

[184] Kier A.B. (1990). Clinical neurology and brain histopathology in NZB/NZW F1 lupus mice. J. Comp. Pathol..

[185] Li Y., Eskelund A.R., Zhou H., Budac D.P., Sanchez C., Gulinello M. (2015). Behavioral Deficits Are Accompanied by Immunological and Neurochemical Changes in a Mouse Model for Neuropsychiatric Lupus (NP-SLE). Int. J. Mol. Sci..

[186] Gao H.X., Campbell S.R., Cui M.H., Zong P., Hee-Hwang J., Gulinello M., Putterman C. (2009). Depression is an early disease manifestation in lupus-prone MRL/lpr mice. J. Neuroimmunol..

[187] Schrott L.M., Crnic L.S. (1996). Increased anxiety behaviors in autoimmune mice. Behav. Neurosci..

[188] Moustafa A.T., Moazzami M., Engel L., Bangert E., Hassanein M., Marzouk S., Kravtsenyuk M., Fung W., Eder L., Su J. (2020). Prevalence and metric of depression and anxiety in systemic lupus erythematosus: A systematic review and meta-analysis. Semin. Arthritis Rheum..

[189] Luciano-Jaramillo J., Sandoval-Garcia F., Vazquez-Del Mercado M., Gutierrez-Mercado Y.K., Navarro-Hernandez R.E., Martinez-Garcia E.A., Pizano-Martinez O., Corona-Meraz F.I., Banuelos-Pineda J., Floresvillar-Mosqueda J.F. (2019). Downregulation of hippocampal NR2A/2B subunits related to cognitive impairment in a pristane-induced lupus BALB/c mice. PLoS ONE.

[190] He Y.Y., Yan Y., Zhang H.F., Lin Y.H., Chen Y.C., Yan Y., Wu P., Fang J.S., Yang S.H., Du G.H. (2016). Methyl salicylate 2-O-beta-d-lactoside alleviates the pathological progression of pristane-induced systemic lupus erythematosus-like disease in mice via suppression of inflammatory response and signal transduction. Drug Des. Dev. Ther..

[191] Karnopp T.E., Freitas E.C., Rieger A., Chapacais G.F., Monticielo O.A. (2022). Higher IgG level correlated with vitamin D receptor in the hippocampus of a pristane-induced lupus model. Clin. Rheumatol..

[192] Wigren M., Nilsson J., Kaplan M.J. (2015). Pathogenic immunity in systemic lupus erythematosus and atherosclerosis: Common mechanisms and possible targets for intervention. J. Intern. Med..

[193] Govoni M., Bombardieri S., Bortoluzzi A., Caniatti L., Casu C., Conti F., De Vita S., Doria A., Farina I., Ferraccioli G. (2012). Factors and comorbidities associated with first neuropsychiatric event in systemic lupus erythematosus: Does a risk profile exist? A large multicentre retrospective cross-sectional study on 959 Italian patients. Rheumatology.

[194] Bortoluzzi A., Scire C.A., Bombardieri S., Caniatti L., Conti F., De Vita S., Doria A., Ferraccioli G., Gremese E., Mansutti E. (2015). Development and validation of a new algorithm for attribution of neuropsychiatric events in systemic lupus erythematosus. Rheumatology.

[195] Timoteo A., Inacio N., Machado S., Pinto A.A., Parreira E. (2012). Headache as the sole presentation of cerebral venous thrombosis: A prospective study. J. Headache Pain.

[196] Sacco R.L., Kasner S.E., Broderick J.P., Caplan L.R., Connors J.J., Culebras A., Elkind M.S., George M.G., Hamdan A.D., Higashida R.T. (2013). An updated definition of stroke for the 21st century: A statement for healthcare professionals from the American Heart Association/American Stroke Association. Stroke.

[197] Devinsky O., Schein A., Najjar S. (2013). Epilepsy associated with systemic autoimmune disorders. Epilepsy Curr..

[198] Stock A.D., Wen J., Doerner J., Herlitz L.C., Gulinello M., Putterman C. (2015). Neuropsychiatric systemic lupus erythematosus persists despite attenuation of systemic disease in MRL/lpr mice. J. Neuroinflamm..

[199] Vivaldo J.F., de Amorim J.C., Julio P.R., de Oliveira R.J., Appenzeller S. (2018). Definition of NPSLE: Does the ACR Nomenclature Still Hold?. Front. Med..

[200] Popescu A., Kao A.H. (2011). Neuropsychiatric systemic lupus erythematosus. Curr. Neuropharmacol..

[201] Tomita M., Holman B.J., Santoro T.J. (2001). Aberrant cytokine gene expression in the hippocampus in murine systemic lupus erythematosus. Neurosci. Lett..

[202] Wen J., Stock A.D., Chalmers S.A., Putterman C. (2016). The role of B cells and autoantibodies in neuropsychiatric lupus. Autoimmun. Rev..

